# Sulfonylurea receptor 1 deficiency alleviates acute lung injury by activating PARP1 in epithelial cells

**DOI:** 10.1016/j.isci.2026.117627

**Published:** 2026-10-05

**Authors:** Xiaojuan Yin, Qiaoqiao Zhu, Li Zhao, Wenqing Ma, Liying Wang, Huiling Li, Chao Wang, Yipin Wang, Lin Xu, Haijun Zhang, Xuerong Wang, Wenbin Huang

**Affiliations:** 1 Department of Pathology, the First Affiliated Hospital, and College of Clinical Medicine of Henan University of Science and Technology, Luoyang 471003, China; 2 Department of Pharmacology, School of Basic Medical Sciences, Nanjing Medical University, Nanjing, Jiangsu Province 210029, China; 3 Department of Thoracic Surgery, the Affiliated Cancer Hospital of Nanjing Medical University & Jiangsu Cancer Hospital, & Jiangsu Institute of Cancer Research, Nanjing, Jiangsu Province 210009, China; 4 Department of Oncology, Zhongda Hospital, Medical School, Southeast University, Nanjing, Jiangsu Province 210009, China; 5 The College of Basic Medicine and Forensic Medicine, Henan University of Science and Technology, Luoyang, Henan Province 471003, China

**Keywords:** acute lung injury, DNA damage repair, glibenclamide, PARP1, sulfonylurea receptor 1

## Abstract

Acute lung injury (ALI) is a critical disease with high mortality, yet its mechanisms remain incompletely elucidated. In this study, we investigated sulfonylurea receptor 1 (SUR1), the regulatory subunit of ATP-sensitive potassium (K_ATP_) channels, in the progression of ALI. We observed elevated SUR1 protein levels in the bronchoalveolar lavage fluid (BALF) of patients with sepsis-associated ALI, as well as in BALF and lung tissues of ALI mouse models. Genetic deletion of SUR1, but not Kir6.2 (the pore-forming subunit of K_ATP_ channels), significantly alleviated ALI induced by cecal ligation and puncture (CLP) or by lipopolysaccharide (LPS) intratracheal instillation. Mechanistically, SUR1 interacted with poly (ADP-ribose) polymerase 1 (PARP1), impairing DNA damage repair and promoting apoptosis and NF-κB (nuclear factor kappa B) activation in bronchial epithelial cells. Notably, glibenclamide, a clinically approved SUR1 inhibitor, significantly alleviated ALI, at least in part, by disrupting the SUR1-PARP1 interaction. Our findings identify SUR1 as a new therapeutic target and highlight the potential repurposing of glibenclamide as a therapeutic drug in ALI.

## Highlights

• SUR1 deficiency protects against experimental acute lung injury in mice
• SUR1 interacts with PARP1 to suppress DNA repair and promote epithelial apoptosis
• Glibenclamide protects lung epithelium by disrupting the SUR1-PARP1 interaction
• SUR1 expression is elevated in human and experimental acute lung injury

## Graphical abstract

**Figure:**
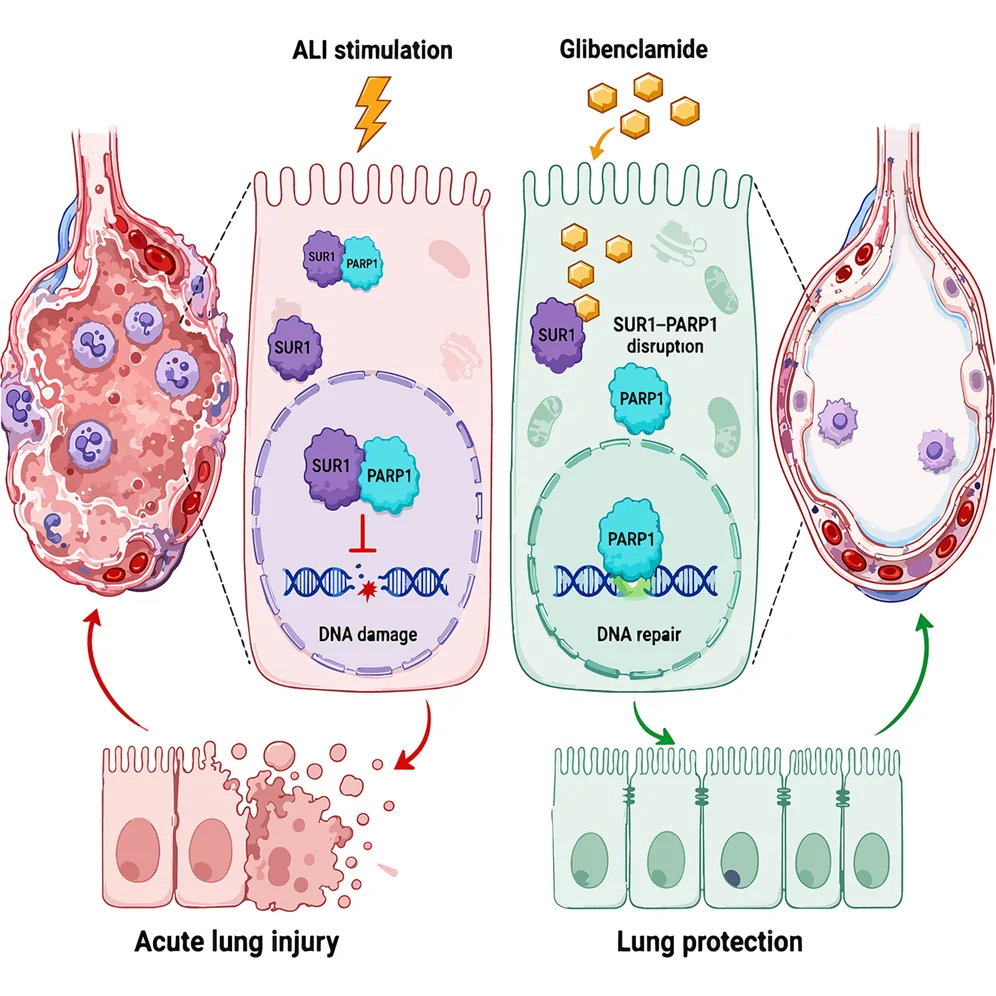

## Teaser

Biological sciences; Molecular biology

## Introduction

Acute lung injury (ALI), a clinical syndrome characterized by tachypnea and hypoxemia, eventually progresses to acute respiratory distress syndrome (ARDS).^1^ ALI/ARDS is a life-threatening condition with an incidence of approximately 7–34 cases per 100,000 people per year and a high mortality rate (60% prior to 2019).^2^^,^^3^ In the post-COVID-19 pandemic era, the incidence of ALI/ARDS has dramatically increased, posing a global public health burden.^4^ While improvements in respiratory critical care management have reduced mortality by 26%–35%, it still remains higher than that of lung cancer, the leading cause of cancer-related death.^1^^,^^5^ The primary clinical interventions for ALI/ARDS include anti-inflammatory drugs, lung-protective mechanical ventilation, and conservative fluid therapy.^6^ Despite these approaches, the low survival rate highlights the urgent need to elucidate the underlying mechanisms of ALI, identify new molecular targets, and develop effective therapies.

ALI is a complex pathophysiological process. Epithelial cells and macrophages are among the first to respond to external or internal insults by releasing cytokines and chemokines, thereby initiating a severe inflammatory cascade involving activation of neutrophils and lymphocytes.^7^ These inflammatory factors cause damage to both alveolar epithelial cells and endothelial cells, and further create a positive feedback loop that exacerbates inflammation and cellular damage.^8^^,^^9^ Among which, epithelial cell damage is not only an initiating factor but also a determinant of disease progression.^10^ Numerous studies have identified critical molecular mediators and elucidated their mechanisms in inflammation, oxidative stress, and cellular apoptosis; however, the precise molecular mechanisms driving epithelial cell injury have not been elucidated.^11^

Sulfonylurea receptor 1 (SUR1) is a regulatory subunit of adenosine triphosphate (ATP)-sensitive potassium channels (K_ATP_ channels) and is primarily known as a therapeutic target for type 2 diabetes mellitus.^12^ In pancreatic β cells, elevated intracellular ATP levels lead to the closure of K_ATP_ channels, resulting in reduced K^+^ efflux, membrane depolarization, Ca^2+^ influx, and subsequent insulin secretion, thereby lowering blood glucose levels.^12^^,^^13^ We previously demonstrated that SUR1 promotes lung cancer progression by interacting with p70S6K and activating its downstream signaling, independent of its classical role in K_ATP_ channel regulation.^14^ Considering that lung cancer originates from alveolar and bronchial epithelial cells and is closely associated with chronic inflammation, we hypothesized that SUR1 may contribute to epithelial injury and inflammatory responses by directly modulating intracellular signaling pathways, beyond its traditional function in K_ATP_ channel regulation.^15^ To date, the role of SUR1 in ALI remains unexplored.

Glibenclamide is a well-characterized inhibitor of SUR1 that mimics ATP to close K_ATP_ channels, thereby stimulating insulin secretion from pancreatic β cells. Clinically, it is an approved hypoglycemic agent for the management of type 2 diabetes mellitus. Emerging evidence has demonstrated that glibenclamide significantly alleviates ALI in various animal models, including oleic acid-, ozone-, radiation-, and ventilator-induced ALI, primarily via its anti-inflammatory effects.^6^^,^^16^^,^^17^^,^^18^ Glibenclamide suppresses key inflammatory signaling pathways such as NLRP3 (NLR family domain containing 3), NF-κB (nuclear factor kappa B), and MAPK (mitogen-activated protein kinase) in macrophages and endothelial cells.^19^^,^^20^^,^^21^ Paradoxically, nicorandil, a K_ATP_ channel opener, has also been reported to cf. protective effects in certain mouse models of ALI.^22^ This apparent contradiction may be attributed to differences in drug targets, tissue selectivity, and off-target effects. Genetic knockout (KO) models targeting individual K_ATP_ channel components may help reveal the mechanism. Notably, the actions of glibenclamide and nicorandil on epithelial cells, which play a central role in the pathogenesis of ALI, remain largely undefined.

Poly (ADP-ribose) polymerase 1 (PARP1) is a constructive enzyme that catalyzes the transfer of ADP-ribose residues from NAD^+^ (nicotinamide adenosine dinucleotide) to target proteins, resulting in the formation of poly (ADP-ribose) (PAR) chains and the release of free PAR polymers or oligomers.^23^ Its primary function is to initiate DNA damage repair. Upon sensing DNA strand breaks, PARP1 recruits DNA repair machinery such as ATM (ataxia telangiectasia mutated) and DNA-PK (DNA-dependent protein kinase) to facilitate homologous recombination (HR) or non-homologous end joining (NHEJ), thereby maintaining genomic integrity and cellular homeostasis.^24^^,^^25^ However, excessive activation of PARP1 can exacerbate oxidative stress, apoptosis, and inflammatory responses, particularly in macrophages, endothelial cells, and neutrophils.^26^ PARP1 has been implicated in the pathogenesis of several pulmonary diseases, including ALI, chronic bronchitis, emphysema, and asthma.^27^ PARP1 inhibitors, originally developed for cancer therapy, have shown protective effects in multiple animal models of ALI.^28^^,^^29^ However, their use may also carry the potential risk of injuring lung epithelial cells as a side effect during ALI treatment. To date, the role of PARP1 in lung epithelial cells during ALI remains poorly explored.

In this study, we found that the SUR1 protein levels were elevated in the bronchoalveolar lavage fluid (BALF) of sepsis-associated ALI patients, as well as in mouse ALI models. Utilizing SUR1 KO and Kir6.2 KO mice, we demonstrated that SUR1 deficiency, but not Kir6.2 deficiency, significantly alleviated ALI in two distinct mouse models, the cecal ligation and puncture (CLP)-induced sepsis-associated ALI mouse model and the lipopolysaccharide (LPS) intratracheal instillation-induced ALI mouse model. Mechanistically, SUR1 was found to form a complex with PARP1, sequestering it and impairing its DNA repair function, thereby promoting DNA damage, epithelial cell apoptosis, and inflammation. Glibenclamide treatment alleviated ALI, at least in part, through disruption of the SUR1-PARP1 interaction. Our findings highlight SUR1 as a potential therapeutic target for ALI.

## Results

### SUR1 protein levels were elevated in the BALF of sepsis-related ALI patients and CLP-induced ALI mouse models

We previously reported that SUR1 expression was significantly higher in human lung cancer tissues than in normal lung tissues. Given that lung cancer is a chronic inflammatory and injury-related disease, we hypothesized that SUR1 expression might also increase in ALI, an acute inflammatory and injury-related disease. For ethical reasons, we were unable to collect lung tissue samples from patients. Therefore, we sought to detect SUR1 protein in dot blots prepared from the BALF of patients with sepsis-related ALI. The SUR1 protein level was significantly higher in the BALF of ALI patients compared to the control group (non-ALI and non-bacterial infection patients). Although SUR1 protein was also detected in serum samples from these patients, no significant differences were observed between the ALI and control groups (Figure 1A). A cytokine screening assay of the BALF and serum samples for 12 inflammation-related cytokines showed significantly higher levels of IL-1β, IL-6, and TNF-α in the BALF of ALI patients than in controls, whereas other cytokines, such as IL-2 and IFN-γ, did not differ significantly (Figure 1B; Figure S1). In the serum samples, no significant differences were found in any of the 12 cytokines between the ALI and control groups (Figure 1B; Figure S1). These results indicate a correlation between ALI and elevated SUR1 protein levels in BALF, alongside increased levels of cytokines such as IL-1β, IL-6, and TNF-α. However, due to that dot blot assays are inherently semi-quantitative and very limited number of samples were examined in this study, these data were intended to provide supportive, rather than definitive, evidence for SUR1 presence in BALF. Further investigations are required to determine the prognostic significance of SUR1 in BALF.

**Figure 1:**
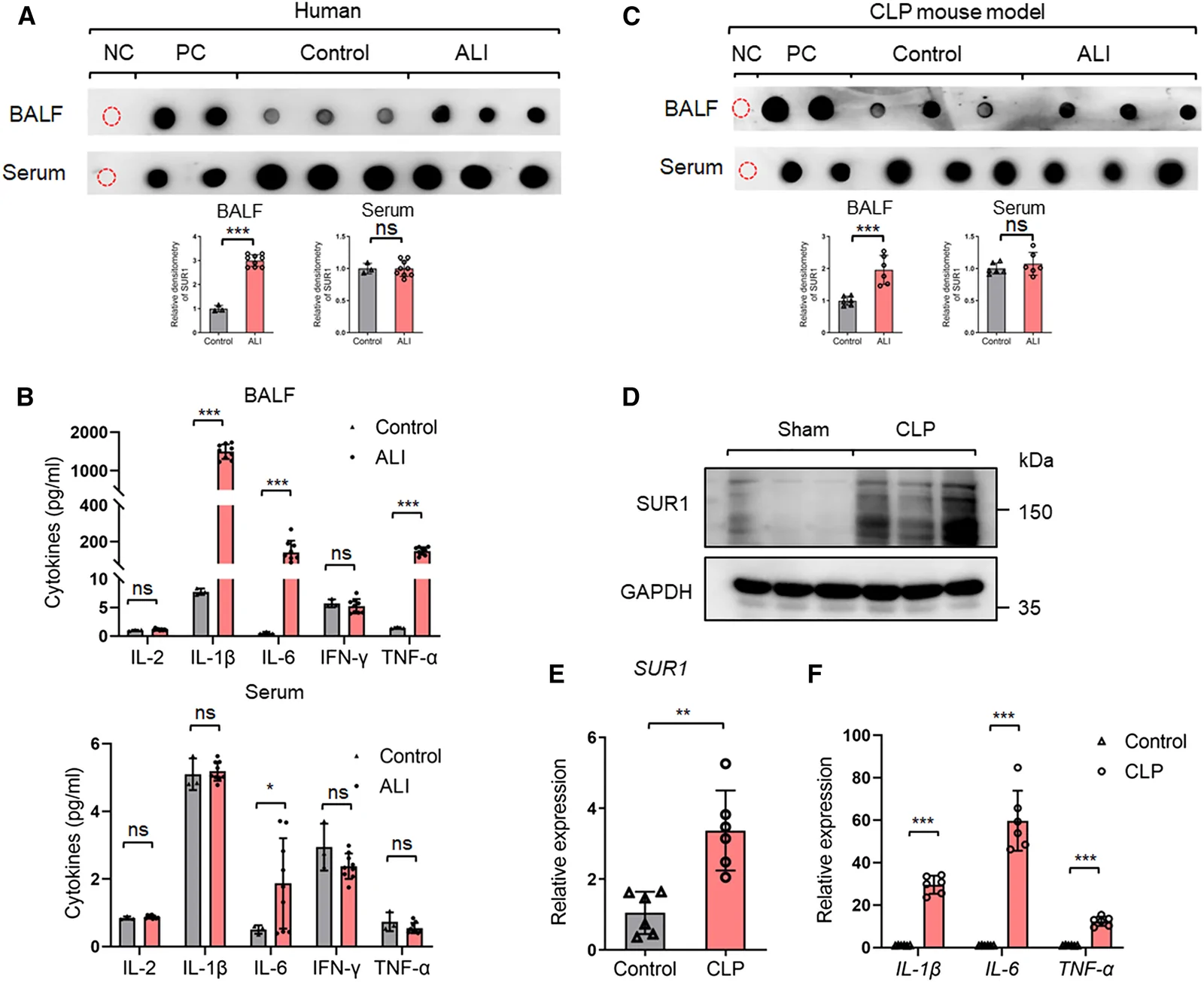
SUR1 protein levels were elevated in the bronchoalveolar lavage fluid (BALF), but not the serum, of sepsis-related ALI patients and CLP-induced ALI mouse models (A) Dot blot analysis of BALF and serum collected from sepsis-related ALI patients or controls. NC (negative control), PBS; PC (positive control), whole-cell protein lysates of HEK293T cells. Control, patients who underwent abdominal surgery without ALI or bacterial infection; ALI, sepsis-associated ALI. Red circle, site of PBS drops. (B) Cytokine levels in BALF and serum of patients determined by a cytokine screening assay. *n* = 3 for control; *n* = 9 for ALI. (C) Dot blot analysis of BALF and serum from CLP-induced ALI mice (*n* = 6). (D–F) Western blot and RT-qPCR analyses of lung tissue samples from CLP-induced ALI mice. Data are presented as mean ± SD. ∗*p* < 0.05; ∗∗*p* < 0.01; ∗∗∗*p* < 0.001; ns, no significance. Data in (A, B, C, and F) were analyzed using Welch’s *t* test, and data in (E) were analyzed using an unpaired two-tailed Student’s *t* test. Representative images are shown.

We then examined the expression of SUR1 in lung tissues from CLP-induced ALI mouse models, which are known to mimic sepsis-associated ALI in humans (Figure S2A). At 24 h after CLP surgery, the mice exhibited clear signs of sepsis, including reduced activity, labored breathing, trembling, and ocular discharge, compared to the sham group. The CLP group mice showed increased lung wet-to-dry weight ratios (wet/dry), indicating pulmonary edema. Hematoxylin and eosin (H&E) staining of lung tissues revealed severe pathological injury, including alveolar destruction, inflammatory cell infiltration, and interstitial edema (Figures S2B and S2C). Evans blue staining also showed a significant increase in vascular permeability in the CLP group (Figure S2D). The total protein content and number of inflammatory cells (especially neutrophils) in the BALF were also markedly higher in the CLP group than in the sham group (Figures S2E and S2F). Consistent with these changes, myeloperoxidase (MPO) activity was significantly greater in lung tissues from the CLP group than in the sham group (Figure S2G), confirming the successful establishment of the CLP-induced ALI mouse model.

We detected SUR1 protein in the serum, BALF, and lung tissues of ALI mice. Dot blotting showed higher levels of SUR1 protein in the BALF of the CLP group than in the sham group; however, no difference was observed in the serum samples between the two groups (Figure 1C). Notably, both SUR1 protein and mRNA levels were significantly higher in the lung tissues of ALI mice compared to those in the sham group (Figures 1D and 1E). In addition, quantitative reverse transcription-polymerase chain reaction (RT-qPCR) assays revealed that the mRNA levels of *IL-1β*, *IL-6*, and *TNF-α* were significantly elevated in the lung tissues of the CLP group relative to the sham group, consistent with the findings from human samples (Figure 1F).

Collectively, these findings suggest that SUR1 protein can be detected in the serum and BALF of both human ALI patients and mouse ALI models. Moreover, the increased levels of SUR1 protein in BALF and lung tissues appear to correlate with the progression of ALI.

### SUR1 knockout alleviated ALI in CLP- and LPS-induced mouse models

We further explored the role of SUR1 in ALI by establishing a SUR1 KO mouse model using CRISPR-Cas9 technology (Figure 2A). Genotyping confirmed wild-type (WT), heterozygous, and homozygous *Sur1* expression (Figure 2B). Western blotting showed that SUR1 protein was undetectable in the SUR1 KO mice (Figure 2C). The phenotypes of SUR1 KO mice (e.g., activity and body weight) did not differ from those of WT mice (Figure S3A). Given that SUR1 is the target of sulfonylurea antidiabetic drugs and regulates insulin release from pancreatic β cells, we also examined diabetes-related markers. SUR1 KO mice exhibited increased fasting and fed blood glucose levels and impaired glucose tolerance compared to WT mice; however, insulin tolerance did not differ significantly between the two groups (Figures S3B–S3E). Thus, our SUR1 KO mouse model showed some aberrant diabetic markers and therefore differed slightly from a previously reported SUR1 KO model.^30^

**Figure 2:**
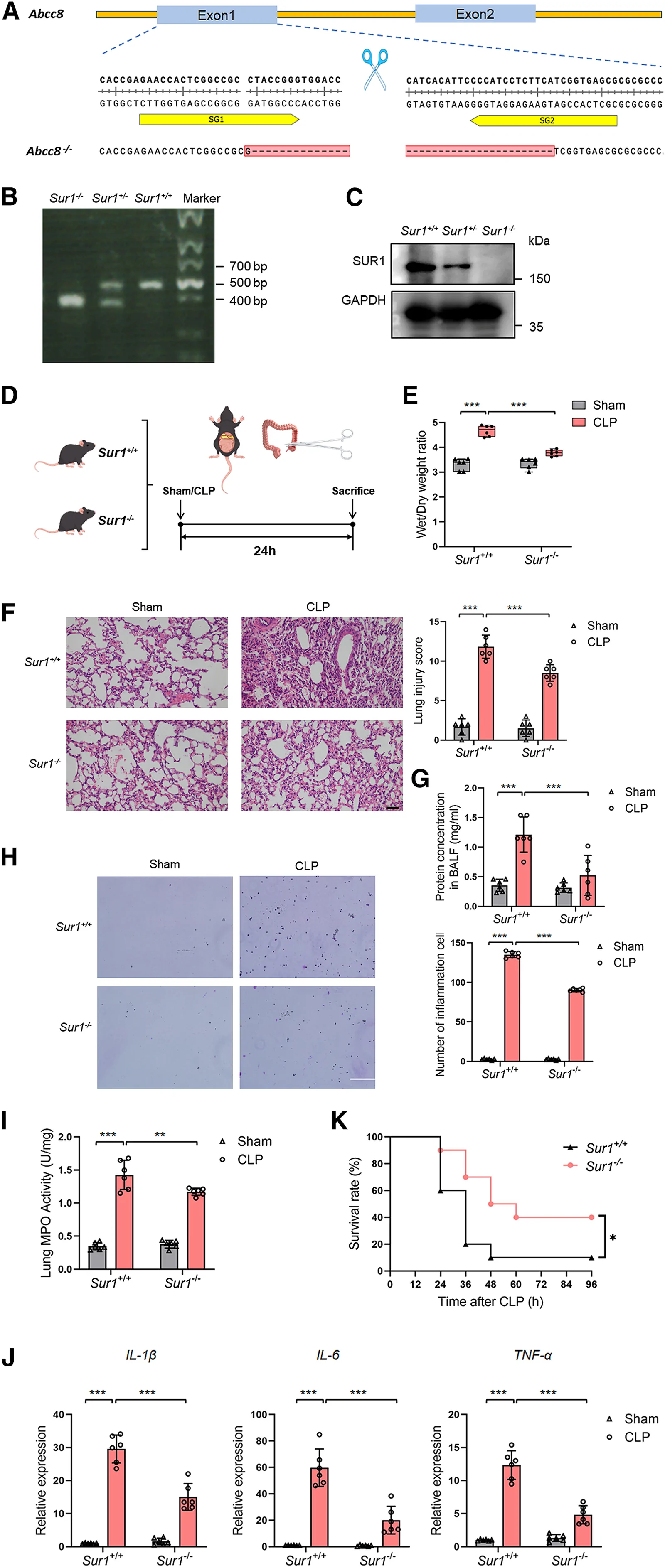
SUR1 knockout alleviated CLP-induced ALI (A) Schematic diagram of SUR1 (*Abcc8*) knockout mouse established by CRISPR-Cas9 technology. SG, sgRNA. (B) Genotyping of SUR1 knockout mice. DNA was extracted from mouse tails and subjected to PCR. PCR products were separated by agarose gel electrophoresis. (C) Western blot analysis of mouse lung tissue samples. (D) Schematic diagram of the CLP-induced ALI mouse model. (E) Lung wet/dry weight ratio. (F) H&E-stained lung sections and injury scores. Scale bars, 50 μm. (G) Protein quantification in BALF. (H) Wright-Giemsa staining of cell smears prepared from BALF, followed by quantification of inflammatory cells. Scale bars, 100 μm. (I and J) MPO activity assay and RT-qPCR assay of lung tissues. (K) Kaplan-Meier survival plots of mouse survival rates (*n* = 10 mice/group). Data are presented as mean ± SD (*n* = 6). Each dot represents an individual mouse. ∗*p* < 0.05; ∗∗*p* < 0.01; ∗∗∗*p* < 0.001; ns, no significance. Data in (E–J) were analyzed using two-way ANOVA followed by Tukey’s multiple-comparisons test. Survival curves in (K) were compared using the log rank (Mantel-Cox) test. Representative images are shown.

We then established the CLP model in SUR1 KO (*Sur1*^−/−^) and WT (*Sur1*^*+/+*^) mice to evaluate the role of SUR1 in sepsis-associated ALI (Figure 2D). CLP-induced ALI was significantly alleviated in SUR1 KO mice, as evidenced by a lower lung wet/dry weight ratio (Figure 2E), reduced pathological injury (as indicated by H&E staining) (Figure 2F), decreased protein content and neutrophil in the BALF (Figures 2G and 2H), reduced MPO activity (Figure 2I), and lower expression of cytokines (*IL-1β*, *IL-6*, and *TNF-α*) (Figure 2J) in KO mouse lung tissues. Notably, the survival rate over a 96-h period following CLP surgery was higher in SUR1 KO mice than in WT mice (Figure 2K). Additionally, no significant differences were observed between SUR1 KO and WT mice in the sham group. Collectively, these findings suggest that SUR1 deficiency significantly alleviates CLP-induced ALI in mouse models.

Subsequently, we conducted examination of the effect of SUR1 deficiency on LPS intratracheal instillation-induced ALI to mimic pneumonia-induced ALI of human beings (Figure 3A). We got similar results, as ALI was significantly alleviated in SUR1 KO mice compared to WT mice. All the measured parameters, including the lung wet/dry weight ratio (Figure 3B), pathological damage (Figure 3C), protein and neutrophil levels in the BALF (Figures 3D and 3E), as well as MPO activity (Figure 3F) and cytokine levels (Figure 3G) in lung tissues, were significantly lower in SUR1 KO mice than in WT mice. These results suggest that SUR1 deficiency significantly alleviates LPS-induced ALI in a mouse model that mimics local pulmonary infection (e.g., pneumonia) associated with ALI.

**Figure 3:**
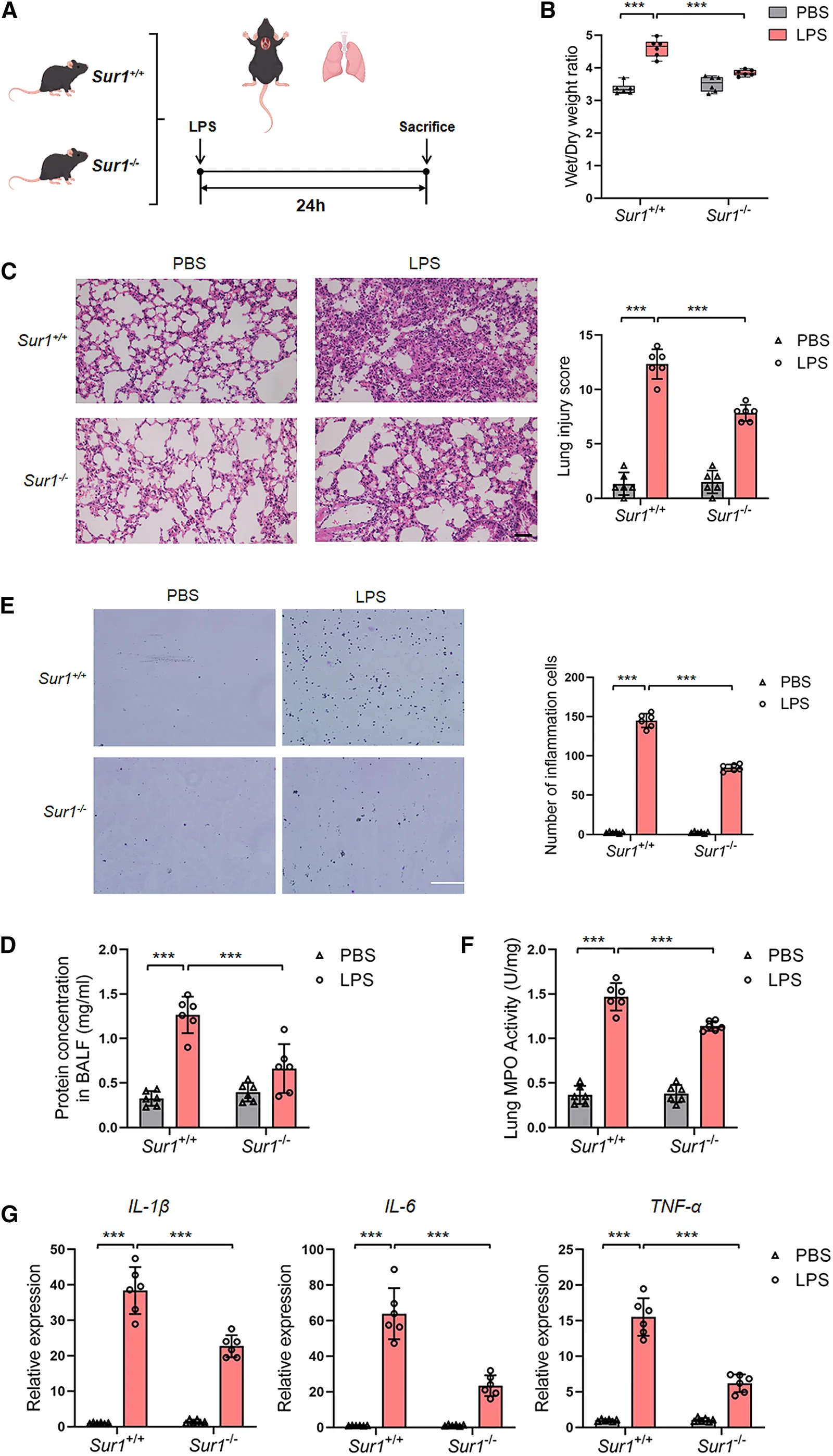
SUR1 knockout alleviated LPS-induced ALI (A) Schematic diagram of the LPS intratracheal instillation-induced ALI mouse model. (B) Lung wet/dry weight ratio. (C) H&E-stained lung sections and injury scores. Scale bars, 50 μm. (D) Protein quantification in BALF. (E) Wright-Giemsa staining of cell smears prepared from BALF, followed by quantification of inflammatory cells. Scale bars, 100 μm. (F and G) MPO activity assay and RT-qPCR assay of lung tissues. Data are presented as mean ± SD (*n* = 6). Each dot represents an individual mouse. ∗∗∗*p* < 0.001. Data in (B–G) were analyzed using two-way ANOVA followed by Tukey’s multiple-comparisons test. Representative images are shown.

### Kir6.2 knockout did not alleviate CLP-induced ALI

Given that SUR1 forms K_ATP_ channels by binding to the pore-forming protein Kir6.2 (encoded by *Kcnj11*) in the lung, we investigated whether the protective effect of SUR1 deficiency in ALI is mediated through its role in the canonical SUR1-Kir6.2 K_ATP_ channel complex. To address this, we utilized Kir6.2 KO mice to examine the contribution of K_ATP_ channels to ALI pathogenesis.^31^ The CLP-induced ALI mouse model (Figure 4A) showed no significant differences in lung wet/dry weight ratios (Figure 4B), pathological lung damage (Figure 4C), protein and neutrophil content in the BALF (Figures 4D and 4E), and MPO activity (Figure 4F) and cytokine levels (Figure 4G) in lung tissues between Kir6.2 KO (*Kir6.2*^−/−^) and WT (*Kir6.2*^*+/+*^) mice.

**Figure 4:**
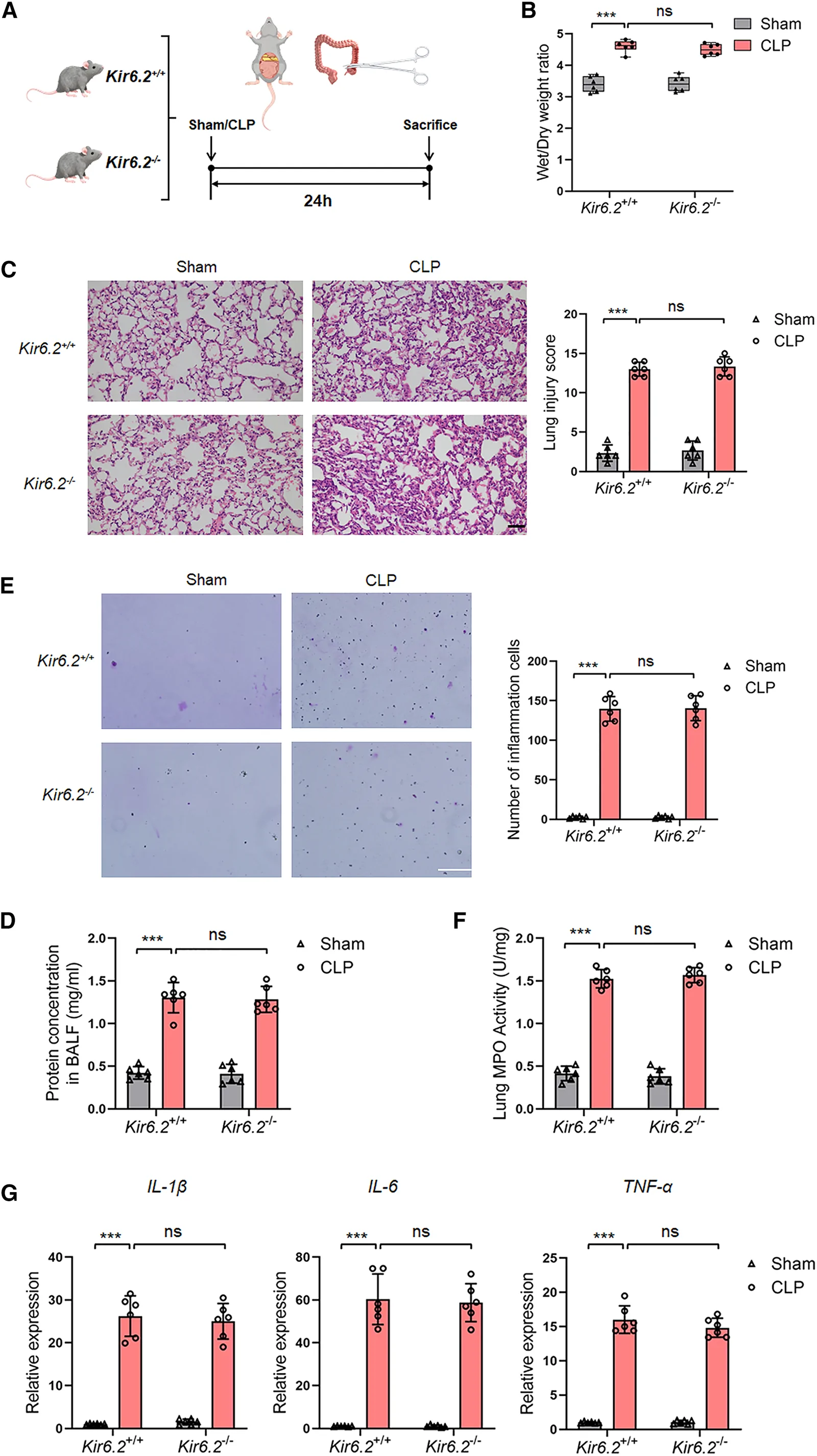
Kir6.2 knockout did not alleviate CLP-induced ALI (A) Schematic diagram of the CLP-induced ALI mouse model. (B) Lung wet/dry weight ratio. (C) H&E-stained lung sections and injury scores. Scale bars, 50 μm. (D) Protein quantification in BALF. (E) Wright-Giemsa staining of cell smears prepared from BALF, followed by quantification of inflammatory cells. Scale bars, 100 μm. (F and G) MPO activity assay and RT-qPCR assay of lung tissues. Data are presented as mean ± SD (*n* = 6). Each dot represents an individual mouse. ∗∗∗*p* < 0.001; ns, no significance. Data in (B–G) were analyzed using two-way ANOVA followed by Tukey’s multiple-comparisons test. Representative images are shown.

Consistently, in the LPS intratracheal instillation-induced ALI model, no significant differences were observed between Kir6.2 KO (*Kir6.2*^−/−^) and WT (*Kir6.2*^*+/+*^) mice (Figure S4). These findings suggest that the canonical SUR1-Kir6.2 K_ATP_ channel complex may not be involved in the SUR1-mediated promotion of ALI.

### SUR1 interacted with PARP1 to suppress DNA damage repair and induce apoptosis of bronchial epithelial cells

To elucidate the molecular mechanism of SUR1 in epithelial cells, we first explored SUR1-interacting proteins by performing co-immunoprecipitation (co-IP) followed by mass spectrometry (MS) analysis in HEK293T cells with FLAG-tagged SUR1 (FLAG-SUR1) overexpression (Table S2). Among the candidates, PARP1 drew our attention, as it is correlated with cell apoptosis and its activation has been implicated in the progression of ALI.^23^ Other candidates, such as eIF3B, vimentin, and PABPC1, are not directly correlated with cell apoptosis, although they showed higher scores than PARP1 (Table S2). We then validated the SUR1-PARP1 interaction by reciprocal co-IP in HEK293T cells, which confirmed mutual binding between the two proteins (Figure 5A). Co-IP in human bronchial epithelial (HBE) cells further confirmed the interaction of endogenous SUR1 and PARP1 (Figure 5A). Moreover, immunofluorescence staining in HBE cells revealed the co-localization of endogenous SUR1 and PARP1 in both the cytoplasm and nucleus, which was supported by a significant Pearson’s correlation coefficient following fluorescence quantification (Figure 5B). Given PARP1’s well-established role in promoting DNA damage repair, we next assessed whether SUR1 affects PARP1 function using a DNA HR reporter assay as previously described.^32^ In this system, successful HR repair restores GFP expression in U2OS-DRGFP cells, which can be quantified by flow cytometry. Under TNF-α stimulation, SUR1 knockdown significantly increased GFP fluorescence intensity compared to control siRNA, indicating that loss of SUR1 enhances PARP1-mediated HR repair (Figure 5C). Furthermore, we examined the phosphorylation of the histone variant H2A.X (γH2AX), a well-established marker of DNA double-strand breaks.^24^^,^^25^ Western blot analysis revealed that both TNF-α and LPS markedly increased γH2AX levels, whereas SUR1 knockdown partially attenuated this effect (Figure 5D). In parallel, phosphorylated p65 NF-κB (p-p65) levels were also reduced upon SUR1 silencing, suggesting that suppression of SUR1 mitigated both DNA damage and inflammatory signaling (Figure 5D). In addition, SUR1 knockdown significantly decreased reactive oxygen species (ROS) generation (Figure 5E) and apoptosis (Figure 5F) in HBE cells in response to TNF-α and LPS stimulation. These findings suggest that SUR1 sequesters PARP1, impeding its role in DNA damage repair and thereby amplifying a pathological feedback loop that promotes ROS accumulation, apoptosis, and inflammation in epithelial cells.

**Figure 5:**
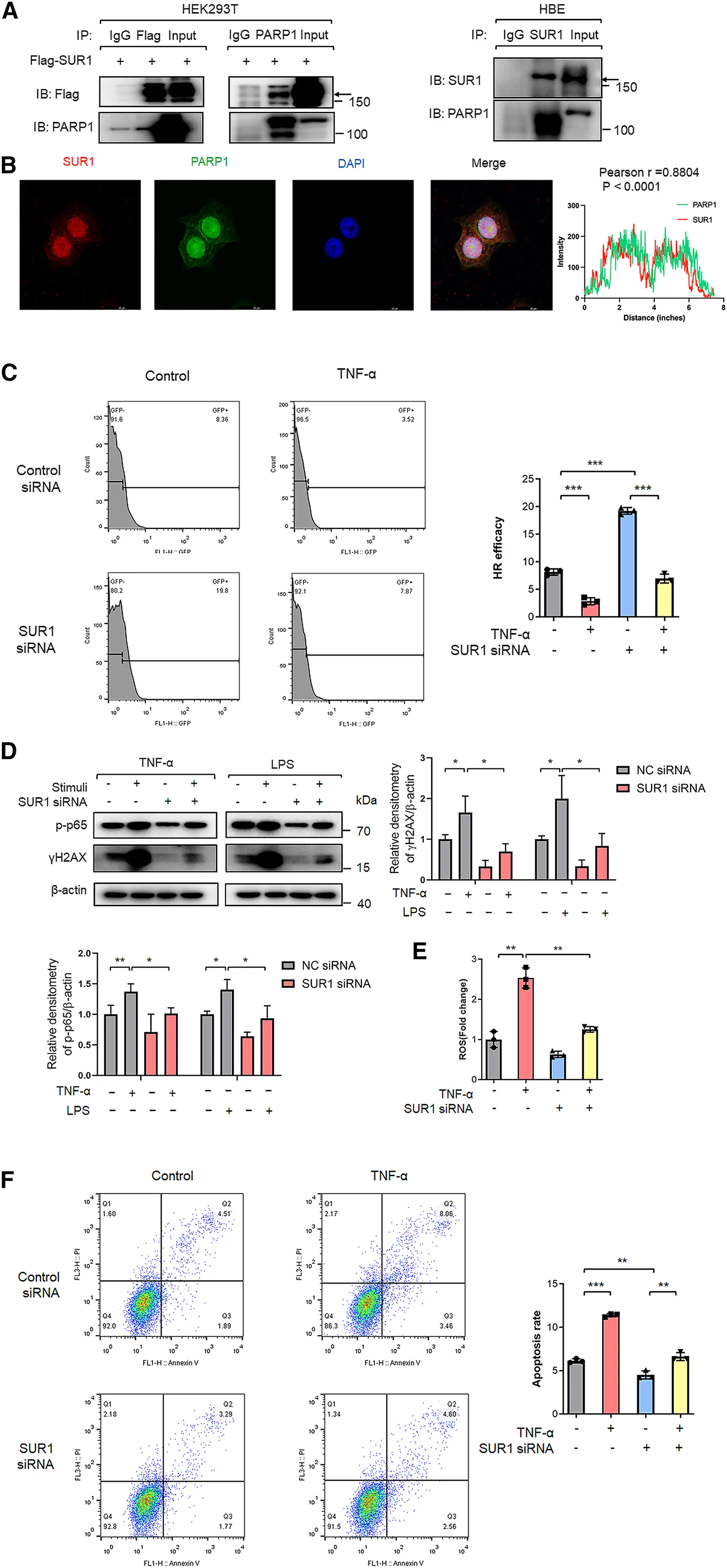
SUR1 interacted with PARP1 to suppress DNA damage repair and induce apoptosis of bronchial epithelial cells (A) Co-immunoprecipitation and western blot analyses of HEK293T cells transfected with FLAG-SUR1 plasmids (left) or human bronchial epithlial (HBE) cells (right). (B) Immunofluorescence staining of HBE cells. Scale bars, 25 μm. (C) Homologous recombination reporter assay in U2OS-DRGFP cells transfected with SUR1 siRNA, followed by flow cytometry analysis. (D) Western blot analysis of HBE cells transfected with SUR1 siRNAs with or without 20 ng/mL TNF-α or 50 μg/mL LPS treatment for 1 h. (E) ROS levels in HBE cells with SUR1 knockdown, treated with or without TNF-α 20 ng/mL for 3 h. (F) Apoptosis assay by flow cytometry in HBE cells transfected with SUR1 siRNAs and treated with TNF-α. Data are presented as mean ± SD (*n* = 3). ∗*p* < 0.05; ∗∗*p* < 0.01; ∗∗∗*p* < 0.001. Co-localization in (B) was assessed using Pearson’s correlation coefficient. Data in (C–F) were analyzed using two-way ANOVA followed by Tukey’s multiple-comparisons test. Representative images are shown.

### Glibenclamide, a SUR1 inhibitor, protected against CLP- and LPS-induced ALI

We then examined the effects of glibenclamide, a SUR1 inhibitor that induces K_ATP_ channel closure, and nicorandil, a K_ATP_ channel opener, in ALI mouse models. Results showed that pretreatment with glibenclamide for 3 days significantly protected against CLP-induced ALI (Figure 6A). Various ALI parameters, such as the lung wet/dry weight ratio, pathological damage, levels of protein and neutrophil in the BALF, and MPO activity and cytokine levels in lung tissues, were all partially reversed in the glibenclamide-treated group (Figures 6B–6G). Moreover, similar protective effects were observed following glibenclamide treatment in LPS-induced ALI mouse models (Figure S5). These findings suggest that glibenclamide protects against ALI.

**Figure 6:**
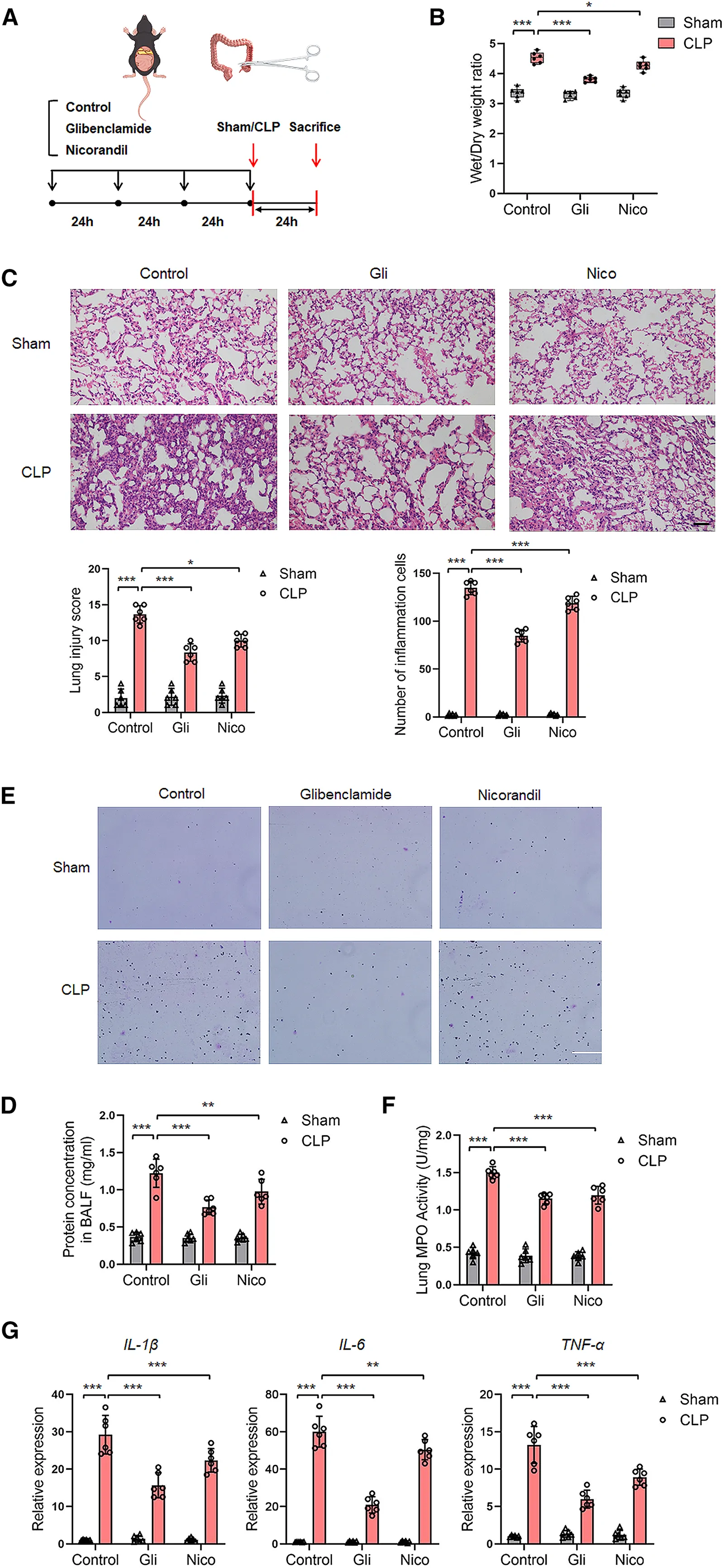
Glibenclamide protected against CLP-induced ALI (A) Schematic diagram of glibenclamide treatment in the CLP-induced ALI mouse model. (B) Lung wet/dry weight ratio. (C) H&E-stained lung sections and injury scores. Scale bars, 50 μm. (D) Protein quantification in BALF. (E) Wright-Giemsa staining of cell smears prepared from BALF, followed by quantification of inflammatory cells. Scale bars, 100 μm. (F and G) MPO activity assay and RT-qPCR assay of lung tissues. Gli, glibenclamide; Nico, nicorandil. Data are presented as mean ± SD (*n* = 6). Each dot represents an individual mouse. ∗*p* < 0.05; ∗∗*p* < 0.01; ∗∗∗*p* < 0.001. Data in (B–G) were analyzed using two-way ANOVA followed by Tukey’s multiple-comparisons test. Representative images are shown.

It should be noted that the K_ATP_ channel opener nicorandil also conferred protection against both CLP-induced ALI (Figure 6) and LPS-induced ALI, but to a lesser extent (Figure S5). Nevertheless, this result was consistent with previous reports.^22^ Given that Kir6.2 deficiency did not cf. protection against ALI and nicorandil has a higher affinity for SUR2 than SUR1, its mechanism of action in this context is likely more complex and may reflect its additional role as a nitric oxide (NO) donor.

Additionally, we performed glibenclamide treatment for CLP-induced ALI in SUR1-deficient (*SUR1*^−/−^) mice. Consistent with our findings in Figure 2, SUR1 KO significantly attenuated lung injury compared with WT controls (*SUR1*^*+/+*^). Notably, glibenclamide treatment still conferred additional protective effects in *SUR1*^−/−^ mice, although the magnitude of this effect was modest (Figure S6). These results suggest that the therapeutic effects of glibenclamide are, at least in part, mediated through SUR1 inhibition but may also involve additional SUR1-independent mechanisms.

### Glibenclamide disrupted SUR1-PARP1 interaction to restore DNA repair and attenuate apoptosis and inflammation in HBE cells

To elucidate the mechanisms underlying glibenclamide’s protective effects in ALI, we performed transcriptome sequencing on HBE cells treated with TNF-α in the presence or absence of glibenclamide, given that epithelial cell homeostasis is critical in both the initiation and progression of ALI (Figure 7A). Given that genes altered by TNF-α and subsequently restored toward baseline levels upon glibenclamide treatment may contribute to its protective effects, we identified differentially expressed genes (DEGs) in both the TNF-α group and the glibenclamide co-treatment group. A total of 280 overlapping genes were identified, as shown in the Venn diagram (Figure 7B). GO-BP (Gene Ontology biological process) and Kyoto Encyclopedia of Genes and Genomes (KEGG) pathway analyses showed that cellular responses to DNA damage, the apoptotic process, and the TNF signaling pathway were significantly enriched (Figure 7C). Further RT-qPCR analysis confirmed that TNF-α downregulated genes involved in DNA damage repair, such as *ATM*, *BRCA1*, *BCL2*, and *PARPBP*, whereas glibenclamide upregulated their expression (Figure 7D). These findings indicate that glibenclamide alleviates TNF-α-induced inflammation and enhances DNA damage repair in bronchial epithelial cells.

**Figure 7:**
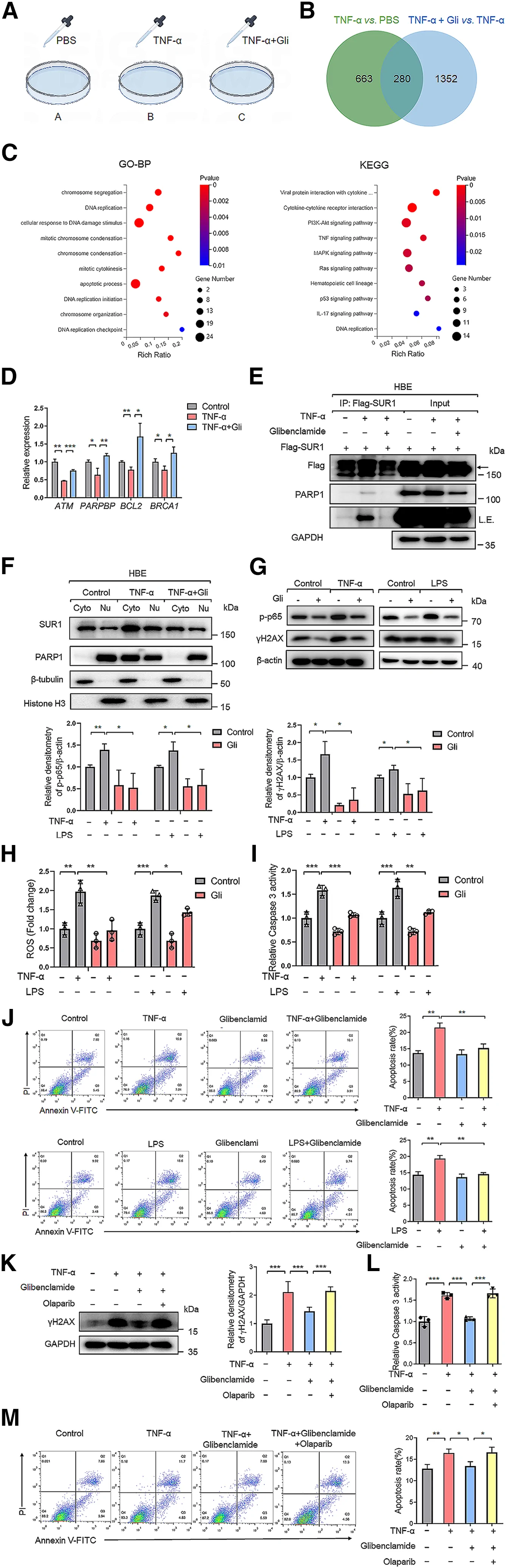
Glibenclamide disrupted SUR1-PARP1 interaction to restore DNA repair and attenuate apoptosis and inflammation in HBE cells (A) Schematic diagram of HBE cells with different treatments, followed by RNA sequencing analysis. Groups: control, 20 ng/mL; TNF-α, 100 μmol/L; glibenclamide plus TNF-α. *n* = 2 per group. (B) Venn diagram of differentially expressed genes. (C) GO and KEGG enrichment analyses of 280 genes in the intersection shown in (B). (D) RT-qPCR analysis of HBE cells with different treatments as indicated. (E) Co-immunoprecipitation assay in HBE cells transfected with FLAG-SUR1 plasmids and treated with 20 ng/mL TNF-α or 100 μmol/L glibenclamide as indicated. L.E., long exposure. (F) HBE cells were treated as in (E), then subjected to cytoplasmic and nuclear protein fractionation followed by western blotting. Cyto, cytoplasm; Nu, nucleus. (G) Western blot analysis of HBE cells pretreated with 100 μmol/L glibenclamide for 30 min followed by TNF-α or LPS co-treatment for 1 h. (H–J) ROS production, caspase-3 activity, and apoptosis in HBE cells under indicated treatments. (K–M) HBE cells were pretreated with 100 μmol/L glibenclamide, 5 μmol/L olaparib, or their combination for 24 h, followed by 20 ng/mL TNF-α stimulation, and subjected to western blotting, caspase-3 activity assay, and apoptosis analysis by flow cytometry. Gli, glibenclamide. Data are presented as mean ± SD (*n* = 3). ∗*p* < 0.05; ∗∗*p* < 0.01; ∗∗∗*p* < 0.001. GO and KEGG enrichment analyses in (C) were performed using a hypergeometric test with false discovery rate (FDR) correction. Data in (D) and (G–M) were analyzed using one-way ANOVA followed by Tukey’s multiple-comparisons test. Representative images are shown.

Given that glibenclamide specifically targets SUR1, we then examined its impact on the SUR1-PARP1 interaction. Co-IP assays showed that TNF-α stimulation enhanced the binding between SUR1 and PARP1, whereas glibenclamide treatment effectively disrupted this interaction in HBE cells (Figure 7E). Cytoplasmic and nuclear fractionation revealed no obvious change in SUR1 localization under either TNF-α alone or TNF-α plus glibenclamide treatment. In contrast, TNF-α induced cytoplasmic accumulation of PARP1, while glibenclamide promoted its predominant nuclear localization (Figure 7F). These findings suggest that SUR1 and PARP1 coexist in both the cytoplasm and nucleus and that glibenclamide disrupts their interaction and alters the subcellular distribution of PARP1.

Moreover, pretreatment with glibenclamide significantly attenuated TNF-α- and LPS-induced increases in γH2AX and p-p65 levels, as detected by western blotting, indicating reduced DNA damage and inflammatory signaling (Figure 7G). Furthermore, glibenclamide significantly decreased TNF-α- and LPS-induced ROS production (Figure 7H), caspase-3 activity (Figure 7I), and apoptosis (Figure 7J). Importantly, the protective effects of glibenclamide were partially reversed by olaparib, a PARP1/2 inhibitor, which restored TNF-α-induced γH2AX accumulation (Figure 7K), caspase-3 activation (Figure 7L), and apoptosis (Figure 7M). These results highlight the critical role of PARP1 in mediating the protective effect of glibenclamide.

These results suggest that glibenclamide disrupts the SUR1-PARP1 interaction, thereby facilitating DNA damage repair and attenuating apoptosis and inflammation in epithelial cells, which may represent one of the mechanisms contributing to its protective effects in ALI (Figure 8).

**Figure 8:**
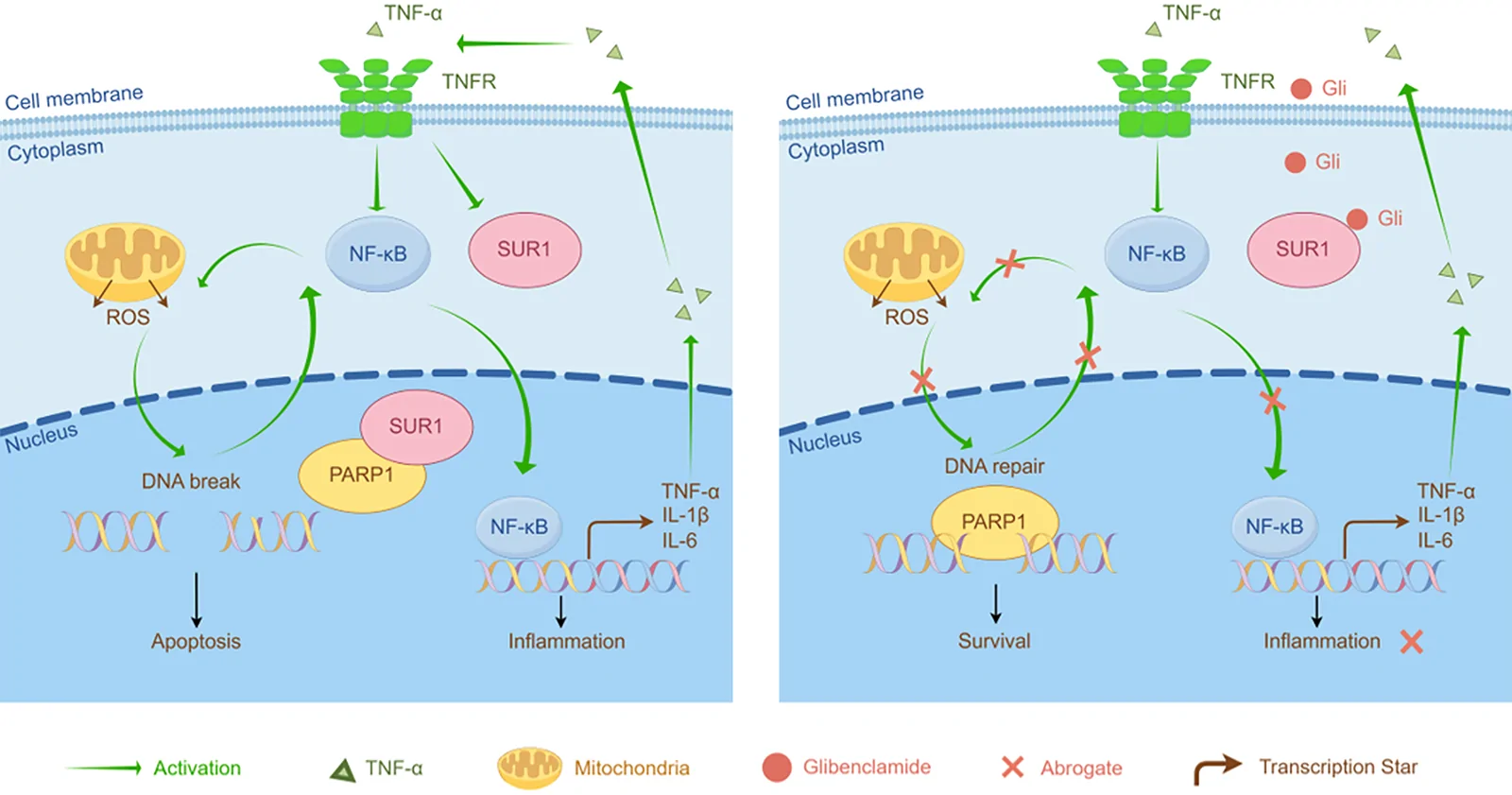
Schematic illustration of the role of SUR1 in ALI and the protective mechanism of glibenclamide TNF-α binds to its receptor (TNFR), activating the NF-κB pathway and promoting inflammation through increased transcription of pro-inflammatory cytokines (IL-1β, IL-6, and TNF-α). In parallel, TNF-α induces mitochondrial dysfunction and ROS production, leading to DNA strand breaks. In response to DNA damage, nuclear PARP1 is activated to recruit DNA repair enzymes, promoting cell survival. Left, SUR1 interacts with and sequesters PARP1, impairing DNA damage repair. Accumulated DNA damage amplifies ROS production and NF-κB activation, which in turn further increases the transcription of inflammatory cytokines. This creates a positive feedback loop that promotes epithelial cell apoptosis and inflammation, contributing to ALI progression. Right, glibenclamide binds to SUR1 and disrupts the SUR1-PARP1 interaction, releasing PARP1 to restore DNA damage repair. This intervention breaks the pathological feedback loop by reducing ROS production, NF-κB activation, and inflammatory cytokine expression, thereby suppressing apoptosis and inflammation in epithelial cells and ultimately protecting against ALI.

## Discussion

ALI is characterized by an inflammatory response, increased microvascular permeability, and damage to the bronchial and alveolar epithelia. However, the mutual activation among leukocytes, endothelial cells, and epithelial cells severely impairs pulmonary structure and function, threatening patients’ lives.^10^ We previously reported the overexpression of SUR1 in lung cancer tissues and its concomitant promotion of the growth and metastasis of lung cancer.^14^^,^^15^ Considering that lung cancer is a chronic inflammatory disease, we suspected that SUR1 might also function in acute lung inflammation, such as ALI, because lung cancer cells originate from normal bronchial and alveolar epithelial cells. In this study, we used CLP- and LPS-induced ALI models to mimic sepsis-associated and pneumonia-associated ALI in humans, respectively.^19^^,^^33^ Our results showed that SUR1 deficiency significantly alleviated ALI, whereas Kir6.2 deficiency had no protective effect. These findings suggest that SUR1 plays an important role in the progression of ALI and that the mechanism may be independent of the canonical SUR1-Kir6.2 K_ATP_ channel complex.

However, several limitations of the current study should be acknowledged, largely due to the use of global SUR1 KO models. First, SUR1 deficiency alters glucose metabolism, which may influence the response to ALI and confound phenotype interpretation. On the one hand, increased glucose availability may enhance cellular tolerance to acute stress, suggesting that hyperglycemia could partially contribute to the protective effect observed in SUR1-deficient mice. On the other hand, hyperglycemia is known to impair host defense and increase susceptibility to infection, which would be expected to exacerbate lung injury. In this context, the protection observed in SUR1-deficient mice may instead reflect a more robust intrinsic effect of SUR1 loss. Notably, Kir6.2 KO mice, which also exhibit abnormalities in glucose metabolism, did not show protection against ALI,^31^ suggesting that altered glucose metabolism is unlikely to be the primary driver of the observed phenotype. Second, other K_ATP_ channel components, such as Kir6.1, SUR2A, and SUR2B, may compensate for the loss of the SUR1-Kir6.2 complex. Therefore, the potential contribution of alternative K_ATP_ channel assemblies to ALI protection cannot be excluded.^34^^,^^35^ Third, the use of global KO models precludes the identification of cell type-specific roles of SUR1 in inflammation and immune responses. As a result, it remains unclear whether the protective effects arise from epithelial, endothelial, or immune cell compartments. Taken together, these limitations highlight that the current model cannot fully disentangle systemic from cell-autonomous effects. Future studies should assess the expression and function of other K_ATP_ channel components across relevant cell types, including epithelial cells, endothelial cells, and macrophages. Moreover, the use of cell type-specific or inducible conditional KO models will be essential to define the precise role of SUR1 in lung injury and to distinguish local pulmonary effects from systemic influences.

Epithelial injury, particularly of bronchial and alveolar epithelial cells, not only initiates but also critically determines the progression and outcome of ALI. However, compared with the well-studied inflammatory responses, the molecular mechanisms of epithelial cell damage remain insufficiently explored. In this study, we focused on the role of SUR1 in epithelial cells during ALI pathogenesis. We found that SUR1 interacts with PARP1 and sequesters it away from DNA damage repair machinery, thereby exacerbating DNA strand break-induced oxidative stress, apoptosis, and NF-κB-mediated inflammation in response to TNF-α or LPS stimulation. This creates a positive feedback loop that amplifies epithelial injury (Figure 8). Previous studies have shown that DNA damage occurs in alveolar type I and type II cells under chronic inflammatory conditions, such as chronic obstructive pulmonary disease (COPD).^36^ Similarly, in acute inflammation, stimuli such as TNF-α, LPS, and environmental toxicants can also induce DNA damage in bronchial epithelial cells.^37^^,^^38^ PARP1 primarily contributes to the repair of DNA damage via the HR pathway, which serves as a direct and efficient survival mechanism for epithelial cells under inflammatory stress.^16^^,^^19^^,^^39^ Accordingly, PARP1 activity at the early stages of ALI is protective by facilitating DNA repair and limiting cell death.^38^ However, excessive or sustained activation of PARP1 at later stages can become detrimental, promoting cell injury via multiple mechanisms, including excessive PARylation of substrate proteins, accumulation of free poly (ADP-ribose) (PAR) polymers, mitochondrial dysfunction, and transcriptional activation of proinflammatory cytokines through NF-κB signaling.^40^ Consistent with this, numerous studies have demonstrated that pharmacological inhibition of PARP1 confers protective effects against ALI in animal models.^26^^,^^29^ In the present study, we showed that SUR1 knockdown enhanced HR repair, reduced γH2AX accumulation, and alleviated oxidative stress and inflammation in HBE cells, suggesting that SUR1 impairs PARP1’s DNA repair function and facilitates early-stage epithelial damage and inflammation during ALI. Although a recent study reported that the PARP1 inhibitor olaparib did not impair DNA integrity in CLP-induced ALI, this discrepancy may stem from the different cellular contexts examined, macrophages in their study versus epithelial cells in ours.^29^ It should be noted that our current data do not distinguish whether the interaction between SUR1 and PARP1 is direct. Co-IP and immunofluorescence analyses suggest that they may associate within a multiprotein complex. Future studies using approaches such as microscale thermophoresis (MST), fluorescence resonance energy transfer (FRET), or surface plasmon resonance (SPR) will be required to clarify whether this interaction is direct. Therefore, elucidating the detailed mechanisms by which SUR1 modulates PARP1 activity and how this interaction contributes to oxidative stress and NF-κB activation in lung epithelial cells is warranted in future.

Glibenclamide is widely recognized for its anti-inflammatory properties in ALI, primarily through its effects on macrophages and neutrophils. In this study, we identified an additional epithelial cell-associated mechanism of glibenclamide action. By targeting SUR1, glibenclamide disrupts the interaction between SUR1 and PARP1, likely through inducing a conformational change in SUR1. This disruption facilitates the release of PARP1, thereby restoring its role in DNA damage repair and protecting bronchial epithelial cells from apoptosis. Notably, glibenclamide also attenuated DNA strand break-induced NF-κB activation, leading to reduced ROS production and downregulation of proinflammatory cytokines, including TNF-α, IL-1β, and IL-6 (Figure 8). Collectively, these findings suggest that glibenclamide protects against sepsis- and LPS-induced ALI, at least in part, by preserving epithelial integrity and suppressing apoptosis via inhibition of the SUR1-PARP1 axis, while also exerting broader anti-inflammatory effects (Figure 8). The identification of nuclear localization of SUR1 is unexpected and expands its potential functional repertoire beyond its established role as a K_ATP_ channel subunit at the plasma and mitochondrial membranes.^41^ Although no apparent nucleocytoplasmic translocation was observed upon TNF-α or glibenclamide treatment, the presence of SUR1 in the nucleus raises the possibility that it may undergo regulated nucleocytoplasmic shuttling. The underlying mechanisms, including potential involvement of nuclear transport machinery, warrant further investigation. Moreover, the precise molecular mechanism by which glibenclamide disrupts the SUR1-PARP1 interaction remains unclear. Although the glibenclamide-binding pocket of SUR1 in the canonical SUR1-Kir6.2 K_ATP_ channel complex has been defined by cryo-EM and functional assays, these binding determinants were identified in the context of channel inhibition and may not fully explain how glibenclamide disrupts the non-canonical SUR1-PARP1 interaction.^42^ Future structural and mutational studies specifically designed for the SUR1-PARP1 complex will be required to define the precise molecular mechanism. Nonetheless, our data demonstrate that glibenclamide reverses TNF-α- and LPS-induced γH2AX expression and that the protective effects of glibenclamide on apoptosis are partially abrogated by the PARP1 inhibitor olaparib, emphasizing the critical role of PARP1 in glibenclamide-mediated DNA damage repair.

The protective effects of glibenclamide have been reported in multiple animal models of ALI, including those induced by oleic acid, ozone, radiation, hemorrhagic shock, and mechanical ventilation.^16^^,^^19^^,^^20^ However, nicorandil, a K_ATP_ channel opener, has also been reported to cf. protection against ALI in certain models.^22^ In this study, we demonstrated that both glibenclamide and nicorandil significantly attenuated sepsis- and LPS-induced ALI; however, glibenclamide exhibited a more pronounced protective effect. This apparently contradictory result may reflect the nonspecific effects of the drugs. For example, nicorandil is also a NO donor, and NO signaling has been reported to activate protein kinase C (PKC), which may contribute to anti-apoptotic effects. Furthermore, nicorandil has antioxidant properties and may reduce mitochondrial dysfunction by limiting excessive ROS generation. It may also inhibit NF-κB activation and downstream inflammatory cytokine production.^43^ Additionally, other K_ATP_ channel subunits, such as Kir6.1, SUR2A, and SUR2B, may contribute to functional diversity and potentially influence the overall channel activity under conditions of SUR1 deficiency or pharmacological inhibition.^34^^,^^35^ The roles of these subunits across different cell types, including epithelial cells, endothelial cells, and macrophages, remain to be determined. Notably, while SUR1 KO mice exhibited significant protection against ALI, Kir6.2 KO mice did not show a similar phenotype. These findings highlight the critical role of SUR1 and suggest that the protective effects of glibenclamide may involve additional mechanisms beyond the canonical SUR1-Kir6.2 K_ATP_ channel complex blockade.

Another limitation of the present study is that the mechanistic link between the *in vitro* findings and the *in vivo* outcomes remains incompletely defined. While our *in vitro* experiments demonstrate that glibenclamide modulates epithelial cell apoptosis through the SUR1-PARP1 axis, the *in vivo* assessments primarily focused on inflammatory parameters, including cytokine production, immune cell infiltration, and histological lung injury. Moreover, systemic administration of glibenclamide likely affects multiple cell types, including both epithelial and immune cells, complicating the interpretation of cell type-specific mechanisms. Notably, the anti-inflammatory effects of glibenclamide have been widely attributed to inhibition of the NLRP3 inflammasome in immune cells, particularly macrophages.^44^^,^^45^ Therefore, our current data do not establish that regulation of epithelial apoptosis is the predominant mechanism underlying the *in vivo* protective effects. Collectively, our findings reveal a novel epithelial-associated mechanism of glibenclamide action, while not excluding the well-established contribution of immune-mediated pathways, such as NLRP3 inflammasome inhibition. Future studies using cell type-specific *in vivo* models will be required to delineate their relative contributions. In addition, hypoglycemia, the major clinical adverse effect of glibenclamide, can be life-threatening and significantly limits its therapeutic use in ALI, particularly in normoglycemic patients. Therefore, developing derivatives that selectively disrupt the SUR1-PARP1 interaction while sparing the SUR1-Kir6.2 complex may represent a safer strategy. Furthermore, PROTAC-based approaches targeting SUR1, which are generally less effective at degrading membrane-associated proteins, may in this context offer a unique advantage by sparing membrane-localized SUR1. This property may help reduce the risk of hypoglycemia and provide a promising avenue for ALI therapy.

Other important findings of this study include the detection of SUR1 protein in the serum and BALF of human ALI patients and the significantly higher levels of SUR1 in the BALF of sepsis-related ALI patients compared to nonbacterial infection or non-ALI patients. It should be noted that we measured soluble SUR1 in the supernatant of BALF and serum rather than SUR1 within intact cells. Therefore, the increased SUR1 protein may originate from damaged or dead epithelial cells or be secreted by epithelial cells, as we found that SUR1 expression was upregulated in ALI. Our ALI mouse models confirmed the correlation between SUR1 protein levels in BALF, lung tissues, and the severity of ALI. BALF serves as a more direct medium for assessing pathological changes in the lung during ALI, as both serum and BALF are considered liquid biopsy samples, which are regarded as non-invasive diagnostic tools. Although we observed increased SUR1 protein levels in the BALF of ALI patients, the prognostic significance of SUR1 in ALI requires further investigation. Future studies should include larger patient cohorts, prospective clinical trials, and the development of ELISA kits for precise quantification of SUR1. Upon further validation, SUR1 may serve as a potential prognostic marker for ALI.

In summary, we found that SUR1 protein levels were elevated in the BALF of patients with sepsis-associated ALI compared to non-ALI controls. Genetic ablation of SUR1 significantly attenuated CLP- and LPS-induced ALI in mouse models. Mechanistically, SUR1 interacted with PARP1 and sequestered it from participating in DNA damage repair, thereby exacerbating ROS production, apoptosis, and inflammation in response to DNA damage. Glibenclamide, by targeting SUR1, disrupts the SUR1-PARP1 interaction, thereby facilitating PARP1-dependent DNA repair and reducing epithelial cell apoptosis and inflammation, representing a potential mechanism contributing to its protective effects in ALI. Together, our findings identify SUR1 as a novel therapeutic target and highlight the potential of glibenclamide as a repurposed drug for the treatment of ALI.

### Limitations of the study

Although this study identifies SUR1 as a regulator of epithelial injury in ALI, several limitations should be acknowledged. First, the number of clinical samples analyzed in this study was relatively limited, and larger patient cohorts will be required to further validate the association between SUR1 expression and disease severity. Second, although SUR1 deficiency and pharmacological inhibition with glibenclamide alleviated lung injury in experimental models, the role of SUR1 was investigated primarily in lung epithelial cells. Potential contributions from other lung-resident cells, particularly immune cell populations, cannot be excluded and should be addressed using cell type-specific KO models in future studies. Third, while our data demonstrate that SUR1 interacts with PARP1 to suppress PARP1-mediated DNA repair, the structural basis of this interaction and the domains responsible for SUR1-PARP1 binding remain to be elucidated. Finally, although glibenclamide showed therapeutic efficacy in preclinical models, its translational potential requires further investigation, including optimization of dosing strategies, therapeutic window, pharmacokinetic properties, and safety before clinical application.

## Resource availability

### Lead contact

Further information and requests for resources and reagents should be directed to and will be fulfilled by the lead contact, Wenbin Huang (wbhuang348912@126.com).

### Materials availability

The SUR1 knockout mouse line used in this study was independently generated using a distinct gene-targeting strategy. This line is available from the lead contact upon reasonable request, subject to an appropriate material transfer agreement and availability of sufficient breeding colonies.

Plasmids generated in this study are available from the lead contact upon reasonable request and completion of an appropriate material transfer agreement.

### Data and code availability

• RNA-seq data generated in this study have been deposited in the Gene Expression Omnibus (GEO): GSE345117. Other data reported in this paper will be shared by the lead contact upon reasonable request.
• This paper does not report original code.
• Any additional information required to reanalyze the data reported in this study is available from the lead contact upon request.

## Acknowledgments

We thank Prof. Gang Hu (Nanjing Medical University) for providing us with the Kir6.2 knockout mice (*Kcnj11*^−/−^). We thank Prof. Guo Chen (China Pharmaceutical University) for providing us with the U2OS-DRGFP cells and the pCBASceI plasmids. This work was funded by the 10.13039/501100001809National Natural Science Foundation of China (grant nos. 81972763 and 81473241 to X.W., grant no. 81872378 to L.X.); a key project of the Science and Technology Development of Luoyang (2302015A) to W.H.; and a Joint Grant of Science and Technology Investigation of Henan Province (242103810042) to W.H.

## Author contributions

X.Y., Q.Z., L.Z., W.M., L.W., H.L., C.W., and Y.W. conducted the experiments; W.H., X.W., H.Z., and L.X. designed the experiments and wrote the paper. All authors read and approved the final manuscript.

## Declaration of interests

The authors declare no conflict of interest.

## Declaration of generative AI and AI-assisted technologies in the writing process

The authors used ChatGPT (OpenAI) solely to assist with English language editing and improve the clarity, grammar, and readability of the manuscript. No artificial intelligence tools were used for data collection, data analysis, interpretation of results, experimental design, or generation of scientific conclusions. All scientific content, figures, conclusions, and final manuscript revisions were reviewed, verified, and approved by the authors.

## STAR★Methods

### Key resources table

**Table:** 

REAGENT or RESOURCE	SOURCE	IDENTIFIER
**Antibodies**		

Anti-Phospho-NF-κB p65 (Ser536) (93H1)	Cell Signaling Technology	Cat# 3033; RRID: AB_331284
Normal rabbit IgG	Cell Signaling Technology	Cat# 2729; RRID: AB_1031062
Anti-SUR1 (ABCC8)	Affinity Biosciences	Cat# DF9254; RRID: AB_2842450
Anti-PARP1	Proteintech Group	Cat# 13371-1-AP; RRID: AB_2160459
Histone H3	Proteintech Group	Cat# 17168-1-AP; RRID: AB_2716755
Anti-β-actin	Bioworld Technology	Cat# BS6007M; RRID: AB_2904238
Anti-GAPDH	Bioworld Technology	Cat# AP0063; RRID: AB_2651132
Rabbit IgG	Bioworld Technology	Cat# BD0051
Anti-Phospho-Histone H2AX-S139	ABclonal Biotechnology	Cat# AP0687; RRID: AB_2863808
Rabbit IgG HRP-conjugated Antibody	R&D systems	Cat# HAF008; RRID: AB_357235
Goat anti-Rabbit IgG (H+L) Highly Cross-Adsorbed Secondary Antibody Alexa Fluor 594	Life Technologies Co., Invitrogen	Cat# A11037; RRID: AB_2534095

**Chemicals, peptides, and recombinant proteins**		

Glibenclamide	Sigma-Aldrich	Cat# G2539
Lipopolysaccharide (LPS)	Sigma-Aldrich	Cat# L2630
Recombinant human TNF-α protein	ABclonal Biotechnology	Cat# RP00001
Recombinant human IL-6 protein	ABclonal Biotechnology	Cat# RP00201
Lipofectamine 2000 Transfection Reagent	Invitrogen	Cat# 11668019
Coomassie Brilliant Blue G250 (C.I.42655)	Sigma-Aldrich	Cat# 1.15444
Evans blue dye	Aladdin Biochemical Technology Co., Ltd.	Cat# E104208
CHAPS	Sigma-Aldrich	Cat# C5070
TRIzol reagent	Invitrogen	Cat# 15596018
Olaparib	Beyotime Biotechnology	Cat# SC9118

**Critical commercial assays**		

MPO Detection Kit	Nanjing Jiancheng Bioengineering Institute	Cat# A044-1-1
ROS Kit	Beyotime Biotechnology	Cat# S0033S
Caspase 3 Activity Assay Kit	Beyotime Biotechnology	Cat# C1115
ABflo 488 Annexin V/PI kit	ABclonal Biotechnology	Cat# RK05875
Nuclear and Cytoplasmic Protein Extraction Kit	Beyotime Biotechnology	Cat# P0028

**Experimental models: Cell lines**		

Human bronchial epithelial cell line HBE	American Type Culture Collection	N/A
Human embryonic kidney cell line HEK293T	American Type Culture Collection	N/A
Human U2OS-DRGFP cells	Gift from Prof. Guo Chen; Chen et al.^32^	N/A

**Experimental models: Organisms/strains**		

SUR1 knockout / *Abcc8*^−/−^ mouse	This paper	N/A
Kir6.2 knockout / *Kcnj11*^−/−^ mouse	Gift from Prof. Gang Hu; Miki et al.^31^	N/A
C57BL/6J mouse	Experimental Animal Center of Nanjing Medical University	N/A

**Oligonucleotides**		

siRNA targeting sequence: SUR1 #1: GUGGUCUACUAUCACAACATT	GenePharma Co., Ltd. (Shanghai, China)	N/A
siRNA targeting sequence: SUR1 #2: GAUCUACCGUCAAAGCUCUTT	GenePharma Co., Ltd. (Shanghai, China)	N/A
siRNA targeting sequence: SUR1 #3: GUCUAUGCCAUGGUGUUCATT	GenePharma Co., Ltd. (Shanghai, China)	N/A
Nontargeting control siRNA: UUCUCCGAACGUGUCACGUTT	GenePharma Co., Ltd. (Shanghai, China)	N/A
qRT-PCR primers used in this study, see STAR Methods	Tsingke Biotechnology	N/A

**Recombinant DNA**		

p3×FLAG-CMV-14-SUR1 / Flag-SUR1 plasmid	This paper	N/A
pCBASceI plasmid	Gift from Prof. Guo Chen; Chen et al.^32^	N/A

**Deposited data**		

RNA-seq data from HBE cells	This paper	GEO: GSE345117

**Software and algorithms**		

ImageJ v1.54f	National Institutes of Health (NIH)	https://imagej.net/ij/
FlowJo v7.6	BD Biosciences	https://www.flowjo.com/solutions/flowjo
GraphPad Prism 8.0	GraphPad Software	https://www.graphpad.com/scientific-software/prism/
SOAPnuke v1.5.6	BGI Genomics	https://github.com/BGI-flexlab/SOAPnuke
HISAT2 v2.1.0	Daehwan Kim Lab	https://daehwankimlab.github.io/hisat2/
RSEM v1.3.1	RSEM developers	https://deweylab.github.io/RSEM/
DESeq2 v1.40.2	Bioconductor	https://bioconductor.org/packages/release/bioc/html/DESeq2.html
BGI Biosys data analysis platform	BGI Genomics	https://biosys.bgi.com/

### Experimental model and study participant details

#### Human participants and sample collection

This study was approved by the Ethics Committee of Nanjing Medical University in accordance with the Declaration of Helsinki (Approval No. 2023-050). Written informed consent was obtained from all participants. Sepsis-associated acute lung injury (ALI) was defined according to the Sepsis-3 criteria (SOFA score ≥2) and the Berlin definition of ARDS. Comprehensive clinical characteristics are provided in Table S1.

#### Animals

Male C57BL/6J mice (8 weeks old, 20–22 g) were purchased from the Experimental Animal Center of Nanjing Medical University. Animals were group-housed (≤5 per cage) under a 12 h light/dark cycle at 22 ± 1°C with free access to standard chow and filtered water. All procedures were approved by the Institutional Animal Care and Use Committee of Nanjing Medical University (IACUC Approval No. 2004038) and conducted in compliance with institutional guidelines. Only male mice were used in this study, sex-specific effects of SUR1 modulation in females were not assessed and represent a limitation for translational interpretation.

SUR1 knockout (*Abcc8*^-/-^) mice were generated by CRISPR-Cas9 targeting the first exon of *Abcc8* on a C57BL/6J background. Two sgRNAs were designed: sgRNA1, 5′-GAACCACTCGGCCGCCTACCGGG-3′; sgRNA2, 5′-CGCTCACCGATGAAGAGGATGGG-3′. Founder mice carried a 104 bp deletion combined with a 1 bp insertion in exon 1, resulting in a frameshift and premature stop codon. Homozygous knockout colonies were established by backcrossing for ≥3 generations. Genotyping primers: forward 5′-AGGGTGCTGCGAGGAACAGA-3′, reverse 5′-TCTGCGGTAGCAAAACTTGG-3′.

The Kir6.2 knockout (*Kcnj11*^-/-^) mice were kindly provided by Prof. Gang Hu (Nanjing Medical University).

#### Cell lines

The human bronchial epithelial cell line HBE and the human embryonic kidney cell line HEK293T were obtained from American Type Culture Collection. U2OS-DRGFP cells, derived from the human osteosarcoma U2OS cell line and carrying a DR-GFP homologous recombination reporter, were generated and kindly provided by Prof. Guo Chen (China Pharmaceutical University). All cells were cultured in Dulbecco’s modified Eagle’s medium (DMEM) supplemented with 10% FBS. Cell lines were authenticated by profiling 20 short tandem repeats, and the cultures were tested periodically using Hoechst staining to exclude mycoplasma contamination.

### Method details

#### Human BALF and serum collection and cytokine screening assays

BALF and serum samples were collected from patients. Among 12 patients, 3 had no evidence of bacterial infection or ALI was classified as the control group. Patients with sepsis-associated ALI were classified as the ALI group (*n*=9), who developed lung injury secondary to various underlying conditions. Cytokine levels in the BALF and serum were quantified using the ABplex Human Cytokine12-Plex Assay Kit according to the manufacturer’s instructions (ABclonal Biotechnology).

#### Dot blot assay

All samples (supernatant of BALF or serum) were dropped onto nitrocellulose membranes at 5 μL per dot. Phosphate-buffered saline (PBS) was used as the negative control. Whole-cell protein lysates of HEK293T cells containing 32 μg protein served as the positive control. The membranes were incubated with anti-SUR1 primary antibody and HRP-conjugated secondary antibody, followed by detection using enhanced chemiluminescence (ECL). Quantification of dot blot signals was performed using ImageJ (v1.54f) software. The intensity of each sample was normalized to that of the HEK293T cell lysate included on the same membrane, which served as a positive control. Relative SUR1 levels were expressed as normalized intensity values. For group comparison, fold changes were calculated by dividing the mean normalized value of the ALI group by that of the control group. Data were presented as column charts. The results should be interpreted as semi-quantitative.

#### Establishment of CLP- and LPS-induced ALI mouse models and glibenclamide or nicorandil treatment

All animal experiments were approved by the Institutional Animal Care and Use Committee of Nanjing Medical University and followed the institutional guidelines. Male C57BL/6J mice (8 weeks old and 20–22 g) were purchased from the Experimental Animal Center of Nanjing Medical University. The animals were fed with appropriate nutrients and filtered water in an optimal temperature and humidity room under a 12 h light-dark cycle.

The cecal ligation and puncture (CLP)-induced ALI model was established as described previously.^33^ Sham-operated mice were treated with the same procedure but without cecum ligation and puncture. For the LPS-induced acute lung injury model, mice were anesthetized and challenged with LPS (3 mg/kg) or PBS (sham group) via intratracheal instillation.^19^ ALI was induced in the mice after 24 hours. Glibenclamide (200 mg/kg) or nicorandil (50 mg/kg) was administered by oral gavage daily for 3 days prior to surgery or LPS exposure.

#### Lung wet/dry weight ratio of mouse

The left lung was weighed immediately after excision (wet weight), then dried in an oven at 50°C for 72 h to obtain the dry weight. The Wet/Dry weight ratio was calculated to assess pulmonary edema.

#### Lung tissue paraffin embedding and H&E staining

The left lung of the mouse was fixed in formalin, embedded in paraffin, sectioned, and stained with hematoxylin and eosin (H&E). Representative images were captured with a scale bar of 50 μm.

#### BALF collection, protein quantification, and cell smears

BALF from the right lung was collected as described previously.^19^ BALF was centrifuged at 300×g for 5 min. The supernatant protein concentration was determined using Coomassie Brilliant Blue G250 (C.I.42655) (1.15444, Sigma-Aldrich) staining. The precipitated cells were resuspended, smeared onto slides, and stained with Wright–Giemsa solution.

#### MPO activity

The right lung of the mouse was collected and assayed for myeloperoxidase (MPO) activity using an MPO Detection Kit (Nanjing Jiancheng Bioengineering Institute, China) following the manufacturer’s instructions. Results were normalized to tissue weight.

#### Evans blue albumin (EBA) pulmonary transvascular flux measurement

The mice were tail-vein injected with 50 mg/kg Evans blue dye (E104208, Aladdin Biochemical Technology Co., Ltd.) 2 h before the end of the experiment. At the end point, the mice were perfused with saline through the right ventricle for 5 min, and the lungs were removed and photographed. The lungs were homogenized, the EBA was extracted using formamide, and the EBA concentration was determined based on OD620 and OD740 values.

#### Gene knockdown and overexpression in cells

SUR1 siRNAs (a pool of 3 sequences targeting SUR1) and a nontargeting control siRNA were synthesized by GenePharma Co., Ltd. (Shanghai, China). SUR1 siRNA sequence 1: 5’ - GUGGUCUACUAUCACAACATT - 3’; siRNA sequence 2: 5’ - GAUCUACCGUCAAAGCUCUTT- 3’; and siRNA sequence 3: 5’ - GUCUAUGCCAUGGUGUUCATT - 3’. The sequence of the nontargeting control siRNA was 5’ - UUCUCCGAACGUGUCACGUTT - 3’. The SUR1 coding sequences were inserted into the p3×flag CMV-14 vector to construct plasmids expressing flag-tagged SUR1 (Flag-SUR1 plasmids). Plasmids and siRNAs were delivered into cells using Lipofectamine 2000 following the manufacturer’s instructions.^14^

#### RNA isolation and quantitative reverse transcription-polymerase chain reaction (qRT-PCR)

Total RNA isolation, qRT-PCR, and data analysis were performed as described previously.^14^ The primers were synthesized by Tsingke Biotechnology, with the sequences below:

Mouse *SUR1*: Forward primer, 5’-CTGCGCTGGATCCTTACCTT-3’,

Reverse primer, 5’-GGATTCTGTCACCCCATCAGA-3’;

Mouse *TNF-α*: Forward primer, 5’-ATCCGCGACGTGGAACTG-3’,

Reverse primer, 5’- ACCGCCTGGAGTTCTGGAA-3’;

Mouse *IL-1β*: Forward primer, 5’-CTACAGGCTCCGAGATGAACAAC-3’,

Reverse primer, 5’-TCCATTGAGGTGGAGAGCTTTC-3’;

Mouse *IL-6*: Forward primer, 5’-GTGCAATGGCAATTCTGATTGT-3’,

Reverse primer, 5’-GGTAGCATCCATCATTTCTTTGTATCT-3’;

Mouse *β-actin*: Forward primer, 5’ -AGATGTGGATCAGCAAGCAG-3’;

Reverse primer, 5’- GCGCAAGTTAGGTTTTGTCA-3’;

Human *GAPDH*: Forward primer, 5’-ATGGGGAAGGTGAAGGTCG-3’,

Reverse primer, 5’-GGGGTCATTGATGGCAACAACAATA-3’;

Human *ATM*: Forward primer, 5’-GCACTGAAAGAGGATCGTAAA-3’,

Reverse primer, 5’-GAGGGAACAAAGTCGGAATAC-3’,

Human *PARPBP*: Forward primer, 5’-AATTGGCGTGCTCTTTGTAA-3’,

Reverse primer, 5’-TCAGTCACGTCCATGTTTTC-3’,

Human *BCL2*: Forward primer, 5’-TTTTAGGAGACCGAAGTCCG-3’,

Reverse primer, 5’-AGCCAACGTGCCATGTGCTA-3’,

Human *BRCA1*: Forward primer, 5’-AATTAGCCGGTCATGGTG-3’,

Reverse primer, 5’-GCTGGAGTGCCGTGGTAT-3’.

#### Western blot and co-immunoprecipitation (co-IP)

Whole-cell proteins were extracted using 1% Triton X-100 lysis buffer, as described previously.^14^ Western blot quantification was performed as follows. For each target protein band (e.g., p-p65 NF-κB, and γH2AX), the intensity was calculated as the ratio of the area value of the target band to that of the housekeeping protein band. Fold changes between treatment and control groups were calculated using the formula: fold change = (value ratio of treatment)/(value ratio of control), and the results were presented as column charts. Data are shown as mean ± SD from three independent experiments.

For immunoprecipitation, the 1% Triton X-100 in the lysis buffer was substituted with 0.5% CHAPS (C5070, Sigma-Aldrich). In brief, 0.5–1.0 mg of cell lysate was subjected to immunoprecipitation, with 30–50 μg of cell lysate serving as the input. Anti-Flag and anti-SUR1 antibodies, along with Protein A/G PLUS-Agarose Immunoprecipitation Reagent (BD0048, Bioworld Technology) were used for the pull-down assays as previously described.^14^

#### Intracellular ROS production and cell apoptosis

ROS production was determined using a ROS Kit (S0033S, Beyotime Biotechnology) following the manufacturer's protocol. Caspase-3 activity to show cell apoptosis was determined using the Caspase 3 Activity Assay Kit (C1115; Beyotime Biotechnology) following the manufacturer’s protocol. Both measurements were performed using a fluorescence microplate reader. Flow cytometry to detect Annexin Ⅴ and Propidium iodide (PI) was used to examine apoptotic cells following manufacturer's protocol of ABflo 488 Annexin V/PI kit (RK05875, ABclonal Biotechnology), and data were analyzed by FlowJo (v7.6) software.

#### Immunofluorescence

Cells were fixed with 4% formaldehyde and permeabilized with 0.2% Triton X-100. Then cells were incubated with primary antibodies, subsequent secondary antibodies, and 4’, 6-Diamidino-2-phenylindole dilactate (DAPI), along with intermittent and appropriate PBS washing. Photographs were collected using confocal microscope. Quantification of red and green fluorescence signals was performed using ImageJ (v1.54f), and the colocalization of SUR1 and PARP1 was assessed using Pearson’s correlation coefficient.

#### Nuclear and cytoplasmic protein separation

Nuclear and cytoplasmic proteins were extracted using a Nuclear and Cytoplasmic Protein Extraction Kit (P0028, Beyotime Biotechnology) according to the manufacturer's protocol.

#### DNA homologous recombination assay

Homologous recombination (HR) efficiency was assessed using a DR-GFP reporter system as previously described.^32^ Briefly, U2OS cells stably expressing the HR reporter substrate (U2OS-DRGFP) contain a nonfunctional GFP gene interrupted by an I-SceI recognition site. Upon I-SceI endonuclease expression, a double-strand break is introduced. The functional GFP can only be restored by HR repair using the downstream iGFP sequence as the template. U2OS-DRGFP cells were transfected with SUR1 siRNAs or control siRNAs, followed by co-transfection with the pCBASceI plasmid to express I-Scel. GFP-positive cells were quantified by flow cytometry, and data were analyzed using FlowJo (v7.6) software.

#### RNA sequencing and bioinformatic analysis

HBE cells were divided into three groups: control, TNF-α, and TNF-α plus glibenclamide groups, with two biological replicates per group. Cells were treated with 20 ng/mL TNF-α alone or in combination with 100 μmol/L glibenclamide for 24 h. Total RNA was extracted using TRIzol reagent (15596018, Invitrogen) and submitted to BGI Genomics Co., Ltd. (Wuhan, China) for RNA quality assessment, library preparation, and sequencing. Poly(A)-containing mRNA was enriched using oligo(dT) magnetic beads and fragmented, followed by cDNA synthesis using random N6 primers. After library construction and quality control, qualified libraries were sequenced on the DNBSEQ platform using single-end 50-bp (SE50) sequencing.

Raw sequencing reads were filtered using SOAPnuke (v1.5.6) to remove adapter-contaminated and low-quality reads. Clean reads were aligned to the human reference genome (hg38) using HISAT2 (v2.1.0), and gene expression levels were quantified using RSEM (v1.3.1). Differentially expressed genes (DEGs) were identified using DESeq2 (v1.40.2), with an absolute log2 fold change (|log2FC|) ≥ 1.5 and a *Q* value < 0.05 as the significance thresholds. The identified DEGs were subjected to Gene Ontology (GO) and Kyoto Encyclopedia of Genes and Genomes (KEGG) pathway enrichment analyses using the BGI Biosys data analysis platform (https://biosys.bgi.com/#/). Enrichment analysis was performed using the hypergeometric test implemented with the phyper function in R, with *p* values adjusted for false discovery rate (FDR). A *Q* value < 0.05 was considered statistically significant.

### Quantification and statistical analysis

Data are presented as mean ± SD. Statistical analyses were performed using GraphPad Prism (version 8.0). Comparisons were performed using an unpaired two-tailed Student’s t-test, Welch’s t-test, one-way ANOVA, or two-way ANOVA, as appropriate. ANOVA was followed by Tukey’s multiple-comparisons test. Survival curves were compared using the log-rank (Mantel–Cox) test. *p* < 0.05 was considered statistically significant.
