# Supplementary material for: Sulfonylurea receptor 1 deficiency alleviates acute lung injury by activating PARP1 in epithelial cells
Source: iScience. 2026 Oct 5;29(10):117627. doi: 10.1016/j.isci.2026.117627 (PMC13673107; doi:10.1016/j.isci.2026.117627)
Supplement: Supplementary file 1 [file mmc1.pdf]

iScience, Volume *29*

Supplemental information

Sulfonylurea receptor 1 deficiency alleviates acute lung injury by activating PARP1 in epithelial cells

Xiaojuan Yin, Qiaoqiao Zhu, Li Zhao, Wenqing Ma, Liying Wang, Huiling Li, Chao Wang, Yipin Wang, Lin Xu, Haijun Zhang, Xuerong Wang, and Wenbin Huang

Supplementary figures

Figure S1

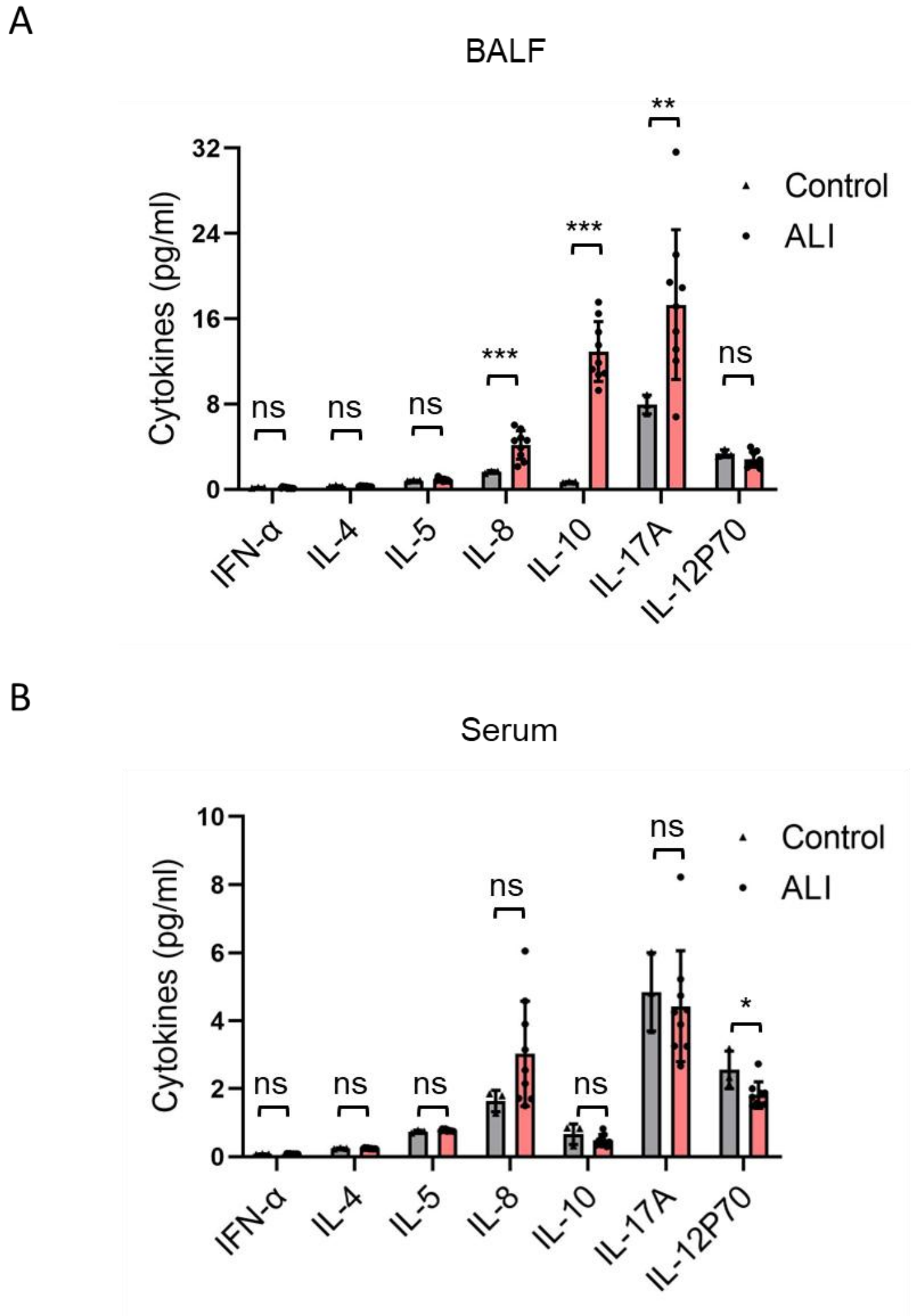

Supplementary Fig. S1 Cytokine levels in the BALF and serum of patients with or without sepsis-related ALI. BALF (A) and serum (B) samples were collected from patients with sepsis-related ALI and control patients (non-bacterial infection and non-ALI patients), and subjected to a cytokine screen assay using ABplex Human Cytokine12-Plex Assay Kit. Data are presented as mean ± SD. n=3 for control; n=9 for ALI. *, $P < 0.05$, **, $P < 0.01$, ***, $P < 0.001$, ns, no significance. Statistical significance was assessed using Welch's t-test.

Figure S2

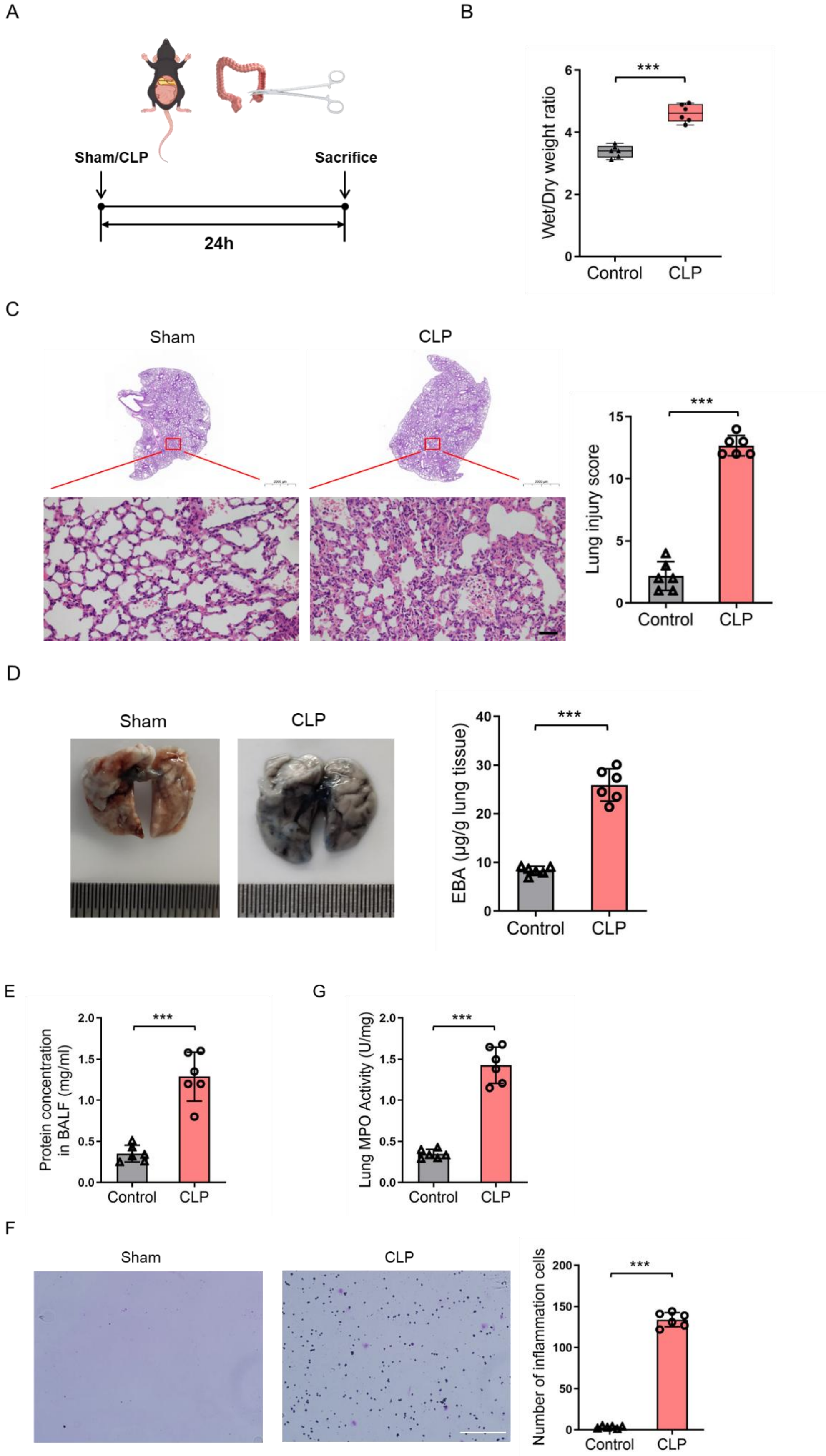
A
Sham/CLP
Sacrifice
24h
B
Wet/Dry weight ratio
***
Control
CLP
C
Sham
CLP
Lung injury score
***
Control
CLP
D
Sham
CLP
EBA (μg/g lung tissue)
***
Control
CLP
E
Protein concentration in BALF (mg/ml)
***
Control
CLP
G
Lung MPO Activity (U/mg)
***
Control
CLP
F
Sham
CLP
Number of inflammation cells
***
Control
CLP

Supplementary Fig. S2 Establishment of a CLP-induced ALI mouse model. A, schematic diagram of the CLP-induced ALI model. n=6 per group. B, lung Wet/Dry weight ratio of mouse. C, H&E-stained lung sections and injury scores. Scale bars, 2000 μm (low magnification) and 50 μm (high magnification). D, Evans blue albumin (EBA) was tail-vein injected 2 h before the mice were sacrificed. Lungs were harvested for imaging, and EBA was then extracted and quantified. E, protein quantification in BALF. F, Wright-Giemsa staining of cell smears prepared from BALF, followed by quantification of inflammatory cells. Scale bar, 100 μm. G, right lung tissues were collected for MPO activity assay. Data are presented as mean ± SD (n=6), each dot represents an individual mouse. ***, $P < 0.001$. Data in B–G were analyzed using an unpaired two-tailed Student's t-test. Representative images are shown.

Figure S3

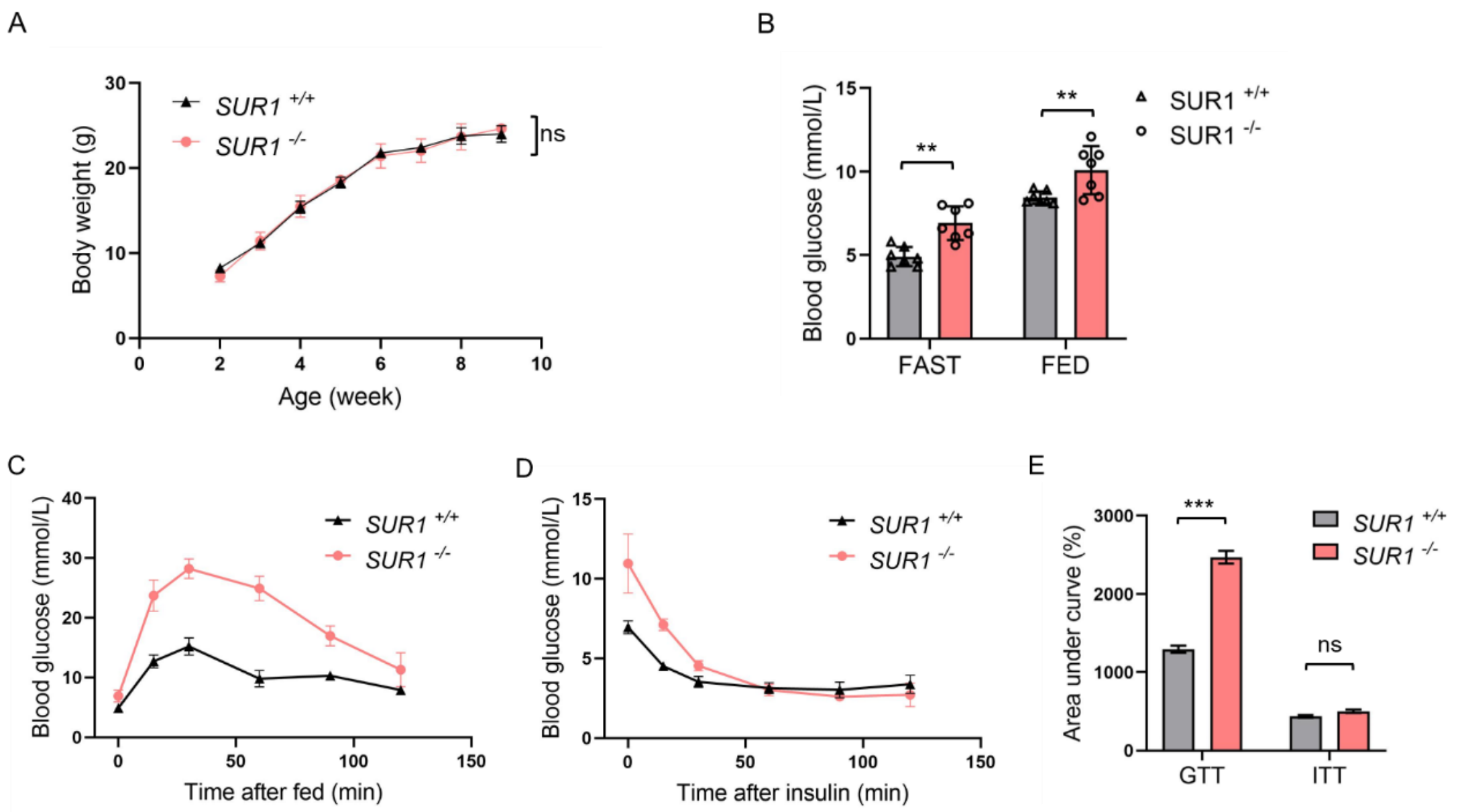

Supplementary Fig. S3 Phenotype of SUR1 knockout mice. A, body weight of SUR1 knockout (*SUR1*$^{-/-}$) and wild-type (*SUR1*$^{+/+}$) mice. B, mice were fasted for 16 h, and blood samples were collected before and after fasting for glucose measurement. C, mice were fasted for 16 h and then administered 2 g/kg glucose by oral gavage. Blood samples were collected at 0, 15, 30, 60, 90, and 120 min after administration for glucose tolerance testing (GTT). D, mice were fasted for 4 h and then administered 0.75 unit/kg insulin by intraperitoneal injection. Blood samples were collected at 0, 15, 30, 60, 90, and 120 min for insulin tolerance testing (ITT). E, quantification of glucose levels in C and D using the area under curve (AUC). Data are presented as mean ± SD, n=6 per group. **, $P < 0.01$, ***, $P < 0.001$, ns, no significance. Data in A, B, and E were analyzed using an unpaired two-tailed Student's t-test.

Figure S4

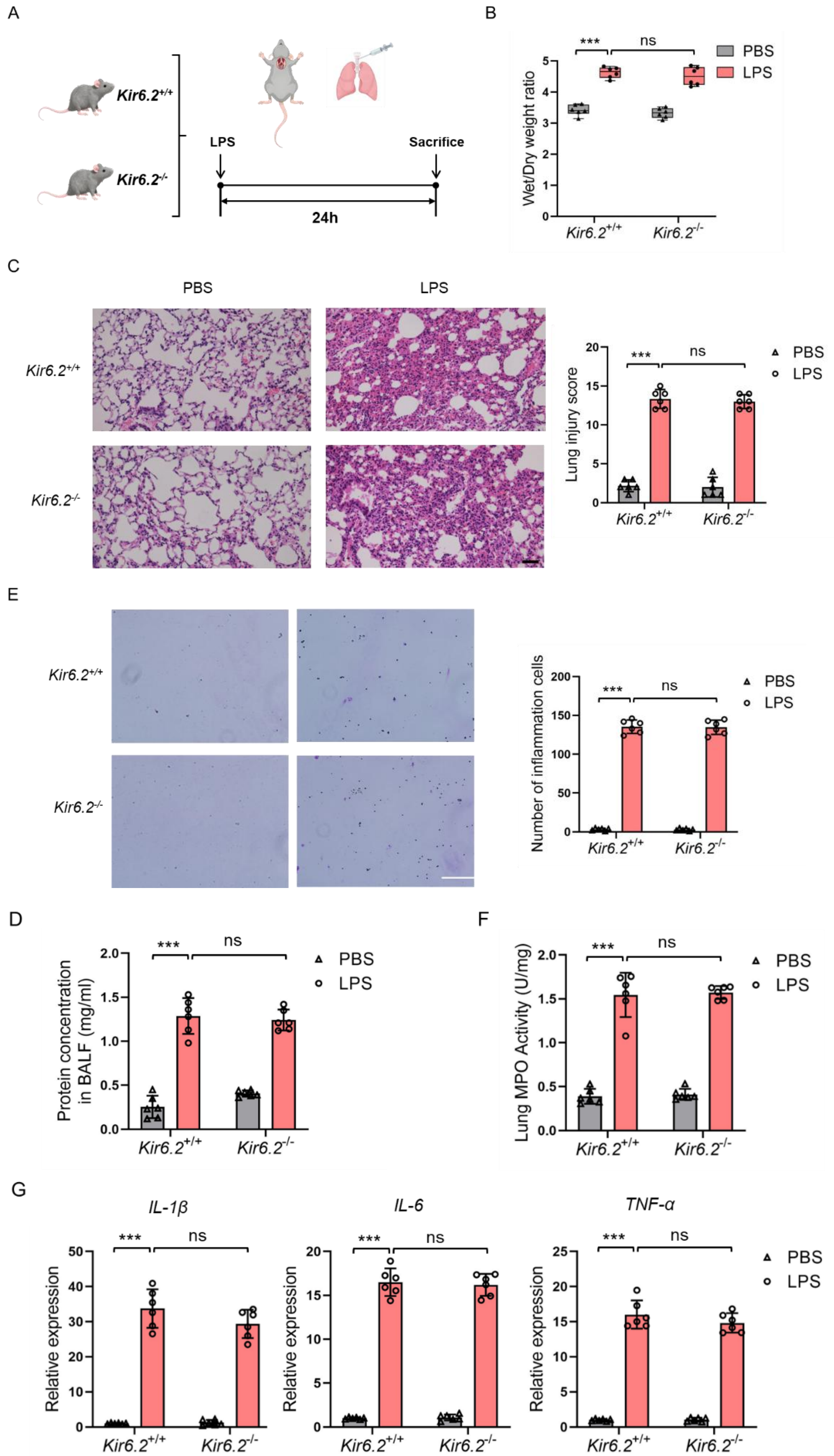
A
Kir6.2+/+
Kir6.2-/-
LPS
Sacrifice
24h
B
***
ns
PBS
LPS
Wet/Dry weight ratio
Kir6.2+/+
Kir6.2-/-
C
PBS
LPS
Kir6.2+/+
Kir6.2-/-
Lung injury score
E
Kir6.2+/+
Kir6.2-/-
Number of inflammation cells
D
Protein concentration in BALF (mg/ml)
F
Lung MPO Activity (U/mg)
G
IL-1β
IL-6
TNF-α
Relative expression

Supplementary Fig. S4 Kir6.2 knockout did not alleviate LPS-induced ALI. A, schematic diagram of the LPS intratracheal instillation-induced ALI mouse model. B, lung Wet/Dry weight ratio. C, H&E-stained lung sections and injury scores. Scale bar, 50 μm. D, protein quantification in BALF. E, Wright-Giemsa staining of cell smears prepared from BALF, followed by quantification of inflammatory cells. Scale bar, 100 μm. F and G, MPO activity assay and qRT-PCR assay of lung tissues. Data are presented as mean ± SD (n=6), each dot represents an individual mouse. ***, $P < 0.001$, ns, no significance. Data in B–G were analyzed using two-way ANOVA followed by Tukey's multiple-comparisons test. Representative images are shown.

Figure S5

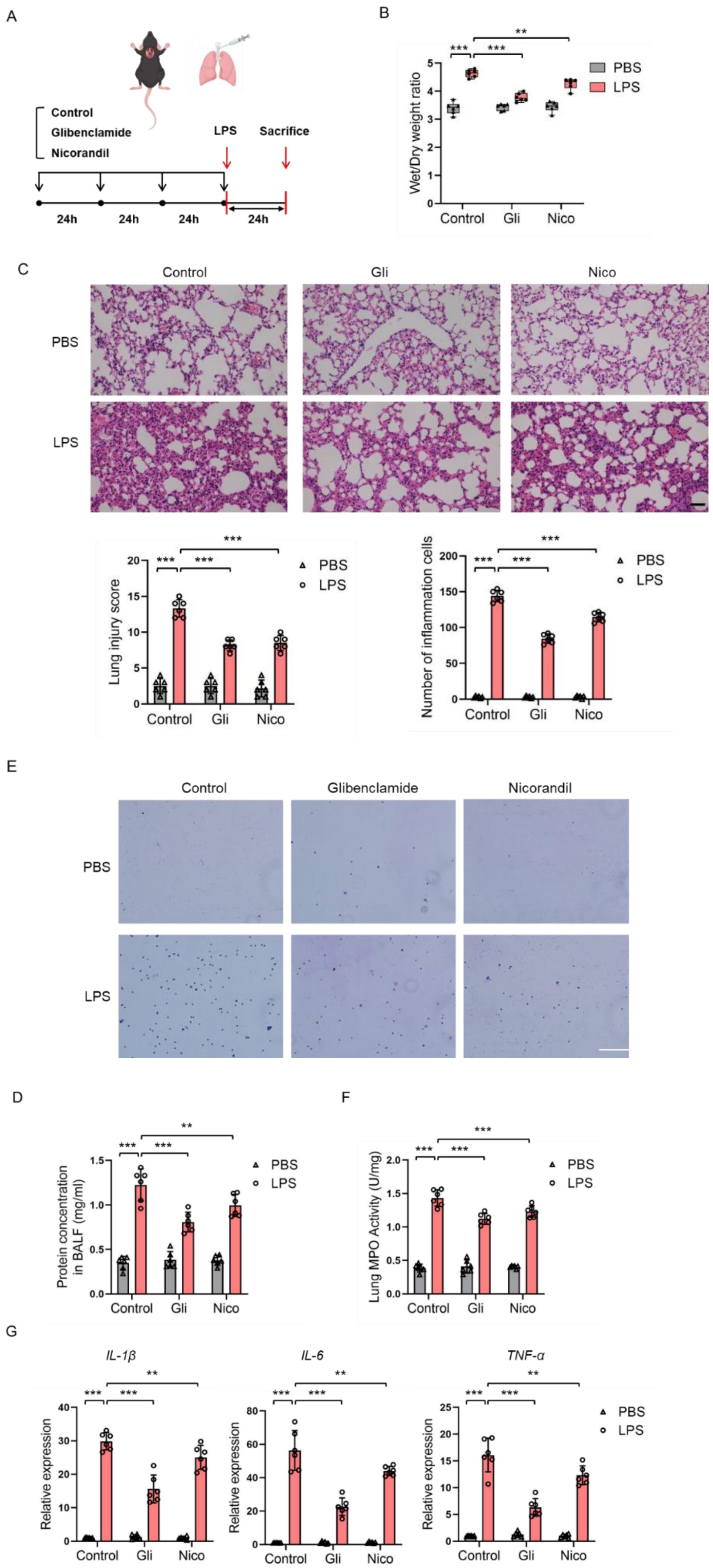
A
Control
Glibenclamide
Nicorandil
LPS
Sacrifice
24h
24h
24h
24h
B
Wet/Dry weight ratio
PBS
LPS
Control
Gli
Nico
C
Control
Gli
Nico
PBS
LPS
Lung injury score
Number of inflammation cells
E
Control
Glibenclamide
Nicorandil
PBS
LPS
D
Protein concentration in BALF (mg/ml)
F
Lung MPO Activity (U/mg)
G
IL-1β
IL-6
TNF-α
Relative expression

Supplementary Fig. S5 Glibenclamide protected LPS-induced ALI. A, schematic diagram of glibenclamide treatment in the LPS-induced ALI mouse model. B, lung Wet/Dry weight ratio. C, H&E-stained lung sections and injury scores. Scale bar, 50 μm. D, protein quantification in BALF. E, Wright-Giemsa staining of cell smears prepared from BALF, followed by quantification of inflammatory cells. Scale bar, 100 μm. F and G, MPO activity assay and qRT-PCR assay of lung tissues. Gli, glibenclamide; Nico, nicorandil. Data are presented as mean ± SD (n=6), each dot represents an individual mouse. **, $P < 0.01$, ***, $P < 0.001$. Data in B–G were analyzed using two-way ANOVA followed by Tukey's multiple-comparisons test. Representative images are shown.

Figure S6

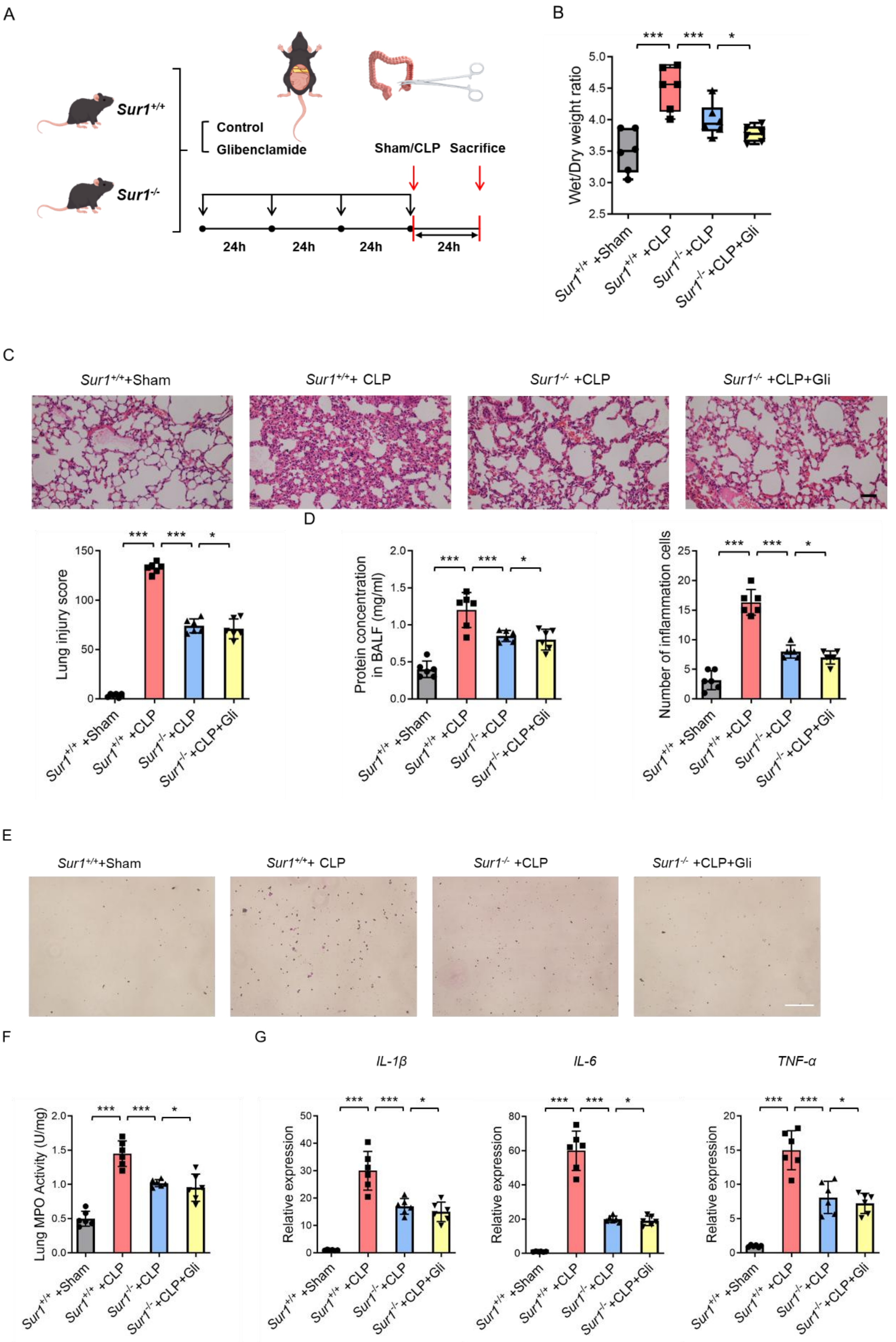

Supplementary Fig. S6 Glibenclamide protected CLP-induced ALI in SUR1 knockout mice. A, schematic diagram of glibenclamide treatment in the CLP-induced ALI in $SUR1^{+/+}$ and $SUR1^{-/-}$ mouse models. B, lung Wet/Dry weight ratio. C, H&E-stained lung sections and injury scores. Scale bar, 50 μm.

D, protein quantification in BALF. E, Wright-Giemsa staining of cell smears prepared from BALF, followed by quantification of inflammatory cells. Scale bar, 100 μm. F and G, MPO activity assay and qRT-PCR assay of lung tissues. Gli, glibenclamide. Data are presented as mean ± SD (n=6), each dot represents an individual mouse. *, $P < 0.05$, ***, $P < 0.001$. Data in B–G were analyzed using two-way ANOVA followed by Tukey's multiple-comparisons test. Representative images are shown.

Supplementary Tables

Supplementary Table S1 The characteristics of patients

Group	Gender	Age	SOFA Score	PaO_2/FiO_2	X-ray	Malignancy
Control	Female	57	1	341.15		Rectal Cancer, Ovarian Cancer
	Female	50	1	376.1		Rectal Cancer
	Female	44	0	448.3		Colon Cancer, Thyroid Cancer
S-ALI	Male	80	4	165.1	Patchy linear hazy opacities in both lungs	Gastric Cancer
	Male	64	5	138.1	Patchy opacities in both lower lungs	Esophageal Cancer
	Male	75	13	147.7	Patchy opacities in the right lung	Esophageal Cancer
	Female	80	9	174.5	Patchy hazy opacities in both lungs	B-cell lymphoma
	Male	76	12	62.4	Patchy opacities in both lower lungs	Gastric Cancer
	Male	67	6	230.4	Patchy hazy opacities in both lungs	Lung Cancer
	Male	84	10	133.0	Patchy and linear opacities in both lungs	Esophageal Cancer
	Male	75	9	97.8	Patchy hazy opacities in both lungs	Lung Cancer
	Male	47	8	94.7	Patchy opacities in both lungs	Esophageal Cancer

SOFA Score (Sequential Organ Failure Assessment): Used to diagnose sepsis. A SOFA score ≥ 2 is one of the core diagnostic criteria according to The Third International Consensus Definitions for Sepsis and Septic Shock (Sepsis 3.0).

PaO_2/FiO_2 (Oxygenation Index): Partial pressure of arterial oxygen (PaO_2) divided by the fraction of inspired oxygen (FiO_2), which is a blood gas parameter of arterial blood.

Supplementary Table S2 The parameters of ABCC8 and PARP1 according to mass spectrometry assay

Gene name	Description	Score	*P* value	Unique peptides	Intensity	iBAQ
ABCC8 (SUR1)	ATP-binding cassette sub-family C member 8	323.31	<0.001	37	3165200000	40975000
PARP1	Poly [ADP-ribose] polymerase	55.761	<0.001	3	21862000	465160
EIF3B	Eukaryotic translation initiation factor 3 subunit B	323.31	<0.001	35	6700600000	130000000
VIM	Vimentin	181.8	<0.001	19	474890000	11412000
PABPC1	Polyadenylate-binding protein 1	76.829	<0.001	8	177390000	3547200

iBAQ (Intensity-Based Absolute Quantification): It is a mass spectrometry-based protein quantification method, primarily used to estimate the absolute abundance of proteins in a sample.
